# Essential and non-essential metals in children’s intellectual functioning: A multi-media biomarker approach from a Mexico City birth cohort

**DOI:** 10.1016/j.envres.2025.122323

**Published:** 2025-07-10

**Authors:** Victor A. Florez-Garcia, Robert O. Wright, Alexander P. Keil, Martha M. Téllez-Rojo, Sandra Martínez-Medina, Guadalupe Estrada-Gutierrez, Amy E. Kalkbrenner

**Affiliations:** a Joseph J. Zilber College of Public Health, University of Wisconsin-Milwaukee, Milwaukee, WI, 53211, United States of America; b Division of Epidemiology and Biostatistics, School of Public Health, University of Illinois Chicago, Chicago, IL, 60612, United States of America; c Department of Public Health, Universidad del Norte, Barranquilla, Colombia; d Department of Environmental Medicine and Public Health, Icahn School of Medicine at Mount Sinai, New York, NY, United States of America; e Epidemiology Branch, National Institute of Environmental Health Sciences, National Institutes of Health, Department of Health and Human Services, Research Triangle Park, NC, United States of America; f Centro de Investigación en Nutrición y Salud, Instituto Nacional de Salud Pública, Cuernavaca, Morelos, Mexico; g Department of Developmental Neurobiology, National Institute of Perinatology, Mexico City, Mexico; h Department of Immunobiochemistry, National Institute of Perinatology, Mexico City, Mexico

**Keywords:** Metals, Cognition, Children, Biomarkers, Mexico, Mixtures

## Abstract

**Background:**

Metal exposures impact children’s intellectual functioning from pregnancy through early childhood and beyond, being historically evaluated with single-pollutant models which might create errors estimating individual metal impacts beyond other correlated metals which arise from the same shared sources.

**Aim:**

We evaluated the effect of exposure to non-essential and essential metals on the cognitive function of Mexican children at 48 months of age.

**Methodology:**

We included persons from the Programming Research in Obesity, Growth, Environment, and Social Stressors (PROGRESS) longitudinal birth cohort in Mexico City with biomarker data on 13 non-essential (lead, cadmium, mercury, arsenic, strontium, barium, and cesium) and essential (manganese, copper, selenium, molybdenum, magnesium, and zinc) metals during pregnancy and early childhood. We assessed the child’s intellectual functioning with McCarthy Scales of Children’s Abilities. We created a multi-media biomarker (MMB) index utilizing the Weighted Quantile Sum Regression (WQS) method. We utilized generalized linear models to estimate the change in the General Cognitive Index (GCI) per interquartile increase in the MMB index(β) and corresponding 95 % confidence intervals(CIs) in single and multipollutant models.

**Results:**

After adjusting for confounders in multipollutant models, MMB-cadmium (β: −1.52, 95 %CI: −3.03 −0.01) and MMB-arsenic (β: −1.53; 95 %CI: −3.02, −0.03) showed significant negative effects on intellectual functioning. In contrast, MMB-manganese (β: 2.28; 95 %CI: 0.77, 3.77), MMB-selenium (β:2.07, 95 %CI: 0.69, 3.43), and MMB-molybdenum (β: 1.76; 95 %CI: 0.21, 3.31) were the metals with the highest positive effects. We did find variations comparing results from single and multi-pollutant models, indicating the presence of multipollutant confounding.

**Conclusion:**

Our study adds to the current body of literature about the co-pollutant confounding problem of evaluating metals and children’s intellectual functioning, as well as the effect of understudied toxic metals of public health interest, such as barium and cesium as candidates that warrant further investigation for a possible role in children’s intellectual functioning.

## 1. Introduction

Proper intellectual functioning is important during childhood because it influences educational achievements, decision-making, and perception of the world around us. The causes of deficits in intellectual function include stressors during pregnancy, (Mandy and Nyirenda, 2018) genetics factors, (Maia et al., 2021) and exposure to environmental pollutants, including metals. (Florez-Garcia et al., 2025; Santa Maria et al., 2019; Vollet et al., 2016) Pregnancy and the first few years of life are critical for neurodevelopment, (Sania et al., 2019; Stiles and Jernigan, 2010) suggesting the importance of exogenous exposures during these periods. Understanding the role of these environmental pollutants on children’s intellectual functioning is relevant because these are usually preventable.

Some environmental causes of cognitive decrements include non-essential (toxic) metals that are well-established developmental neurotoxicants, such as arsenic (As), cadmium (Cd), lead (Pb), and mercury (Hg). (Calderón et al., 2001; Cao et al., 2010; de Water et al., 2022; Dzwilewski and Schantz, 2015; Florez-Garcia et al., 2023; Gustin et al., 2018; Liu et al., 2018) These non-essential metals can cause a reduction in intelligence scores, even at lower levels of exposure. (Braun et al., 2012; Grandjean and Landrigan, 2006; Hubbs-Tait et al., 2009; Kippler et al., 2012, 2016; Lanphear et al., 2005; Lee et al., 2021; Liu et al., 2019; Sanders et al., 2015; Skogheim et al., 2021; Tian et al., 2009) In contrast, other metals are essential components of diet because they are required for human physiology, including, for the development of the central nervous system: copper (Cu), selenium (Se), zinc (Zn), and magnesium (Mg). (Egwunye et al., 2023; Polanska et al., 2016; Warthon-Medina et al., 2015; Wu et al., 2018; Zhou et al., 2020) Yet some essential metals can be harmful when their levels fall outside the normal range. For instance, manganese (Mn) shows an inverted U-shape effect (non--monotonic dose-response), where low levels and high levels are deleterious, but moderate levels support optimal cognitive development. (Bauer et al., 2020; Haynes et al., 2015) Less is known about several other metals that are less studied in relation to human cognitive development, such as cesium (Cs), barium (Ba), molybdenum (Mo), and strontium (Sr). While some epidemiological studies of these metals and human brain function exist, they are limited, and there is contradictory evidence; for instance, one study suggests that higher levels of barium in pregnancy are deleterious to intellectual function scores in children, (Tong et al., 2023) while another points to a positive association between barium and cognition. (Li et al., 2020)

In Mexico, lead-glazed pottery, important in Mexican culture, represents the main source of lead, (Caravanos et al., 2014; Dignam et al., 2019; Téllez-Rojo et al., 2019) which is a recognized neurotoxicant, but is also a potential source of other metals that are used as pigments or for adding physical properties, including zinc, magnesium, strontium, and barium. (Arrevilllaga et al., 2006; Torres, 2009; Vazquez, 2005; Viniegra and Escobar, 1966) Thus, glazed pottery is our main target source of metals as a whole because the potential effect of other metals from this source, and analyzing it may help to better understand not only the negative impact of lead on cognition but also their mixture with other elements from the same source.

We sought to determine the role of individual toxic and nutrient metals in contributing to intellectual functioning in a given context, here, Mexico City, which has a backdrop of concerns about continuing lead exposure from glazed pottery. (Bautista-Arredondo et al., 2023; Caravanos et al., 2014) This assignment is complicated due to the simultaneous presence of multiple metals, making it difficult to identify the effect of each one independently of the others. This phenomenon, known as co-pollutant confounding (i.e.: two pollutants considered at the same time) or multipollutant confounding (i.e.: more than two pollutants considered), is a common challenge in environmental epidemiology research. (Braun et al., 2016; De Rosa et al., 2004) If neglected, the wrong pollutant could be identified as the “bad actor”, leading to interventions that are a waste of resources. Another potential consequence of ignoring co-pollutant confounding is that a beneficial or negative influence of a metal would be missed if its association is masked due to confounding by other metals. Examining two or more metals in statistical models can be used in environmental health sciences to partially solve these confounding issues, and this framework is consistent with recommendations for mixtures analysis. (Stafoggia et al., 2017)

Indeed, there is a growing body of literature about multiple metals, that is, metal mixtures and neurodevelopment. (Bauer et al., 2020; de Water et al., 2022; Rosa et al., 2024; Thilakaratne et al., 2023) An example is a study by Yorifuji et al. in the Faroe Islands, who addressed co-pollutant confounding between lead and mercury on neurodevelopment, finding none. (Yorifuji et al., 2011) Yet the preponderance of metal mixtures literature investigates and reports on interaction (modification), largely neglecting the possibility of co-pollutant confounding, as reviewed by Sanders et al. (2015) Even in the few instances where studies create multi-pollutant models (for example, see Gustin, (Gustin et al., 2018) Kim,(Y. Kim et al., 2013) Zhou),(Zhou et al., 2020) alterations in the measures of association, that is, confounding, are not explicitly addressed with interpretation. Another limitation of this body of literature is that multi-metal models are often limited to 2–3 metals (as in Gustin,(Gustin et al., 2018) Kim,(Y. Kim et al., 2013) and Yorifuji (Yorifuji et al., 2011)). An exception is the publication by Zhou, which did tackle 8 toxic and nutrient metals included simultaneously in models, but again, did not emphasize co-pollutant confounding, perhaps due to their reduced statistical precision in their models including multiple correlated metals, complicating inference. And even though these few studies that have tackled co-metal confounding influencing child cognition have not found much evidence of it, several lines of reasoning supports its importance: a) shared sources creating metal correlations, supporting potential confounding structure, b) that each pair or cluster of metals, including across toxicant and nutrient metals, may have different confounding patterns, calling for further investigation, and c) the importance of considering unique geographic contexts.

Here, we seek to contribute to the understanding of the relationship between metal exposures during important periods of neurodevelopment with childhood intellectual function. The primary objective of our study was to evaluate the effect of exposure to 7 non-essential metals in combination with 6 essential metals in Mexican children in a prospective cohort study measuring neurodevelopment at 48 months of age. To reflect the individual contributions of each metal within the mixture independently of other shared sources, we adjusted each metal for all others. Although there are standard biomarkers to assess the burden of exposure for some metals (e.g, blood lead levels), new methodological mixture approaches to potentially better represent these exposures, combining two or more available biomarkers. (Levin-Schwartz et al., 2020) We also considered toxic metals in the context of nutrient metals and vice versa, tackling a broad range of metals.

## 2. Methodology

### 2.1. Study population and design

Data for the current study come from children from Mexico who were part of the Programming Research in Obesity, Growth, Environment, and Social Stressors (PROGRESS) longitudinal birth cohort in Mexico City. For this study, we excluded 447 subjects without cognitive assessment, and three without metal measurements; thus, we included 604 persons who had the outcome measurement and metal biomarkers, out of 1054 possible participants. (Fig. S1) This cohort has been described in detail previously. (Burris et al., 2013) Briefly, the study enrolled pregnant women who received prenatal care through the Mexican Social Security System (Instituto Mexicano del Seguro Social – [IMSS]) between July 2007 and February 2011. To be eligible, these women had to be < 20 weeks’ gestation, be at least 18 years old, have completed primary education, be without plans to move from Mexico City for the coming 3 years, possess a telephone, not have a medical history of heart or kidney disease, refrain from daily alcohol consumption, and not use steroid or anti-epilepsy medications. (Burris et al., 2013) This study was approved by the institutional review boards at the Harvard School of Public Health, the Icahn School of Medicine at Mount Sinai, the Mexican National Institute of Public Health, and the National Institute of Perinatology (Mexico). This current study was conducted under the oversight of the University of Wisconsin-Milwaukee Institutional Review Board, which granted an exemption due to the use of de-identified data.

### 2.2. Metals exposures

#### 2.2.1. Metals of interest

We are interested in multiple metals as the primary exposures of interest. While the PROGRESS study collected data on 21 metals, in the current study we leverage PROGRESS data on metals that have been previously related to cognition in human epidemiologic literature, whether improving (Krężel and Maret, 2017; Lakshmi Priya and Geetha, 2011; Robberecht et al., 2020; SCSEDRI, 1997) or worsening(Al-Saleh et al., 2020; Braun et al., 2012; Dickerson et al., 2016; Heng et al., 2022; Rodríguez-Barranco et al., 2016; Tolins et al., 2014; Weitzman et al., 1993) cognition when these exposures occurred during prenatal or postnatal time periods. Metals were classified as “unknown evidence” when a limited number of studies were identified, reflecting a limited evidence base rather than a definitive absence of association. Furthermore, we included those metals that have not been well-studied for developmental neurotoxicity but share sources with neurotoxic metals, because metals that share sources may be correlated and lead to confounding. (ATSDR, 2007) We used prior evidence of shared source (e.g. included in Mexican pottery and glazes) and also correlations observed in our data (r > 0.7) (Fig. S2, Table S1). These criteria resulted in including the non-essential metals and metalloids (Lead, Cadmium, Mercury, Arsenic, Strontium, Barium, Cesium, Molybdenum, and Manganese) and essential metals (Copper, Selenium, Magnesium, and Zinc).

#### 2.2.2. Timing and measurement of metals

PROGRESS has multiple measures of each metal of interest during pre- and early post-natal periods. To best capture exposure over this period, we included all available biomarkers of these metals during pregnancy and prior to the measurement of our outcomes (Table S1). We included blood samples collected from pregnant women during the second and third trimesters and at delivery from both cord and maternal venous blood samples, and the child’s blood samples at 12, 24, and 48 months. We included maternal urine samples collected based on two spot urine samples during the second and third trimesters. We also included maternal toenails and hair samples collected during the second and third trimesters (Table S2).

Study staff collected blood samples in facilities affiliated with IMSS using trace metal vacutainer tubes containing EDTA. Then, the blood samples were refrigerated at 4 °C until they were shipped to the trace metals laboratory at the Icahn School of Medicine at Mount Sinai, where they were subsequently stored at −20 °C until analysis using graphite furnace atomic absorption spectrophotometry (Model 3000: PerkinElmer, Wellesley, MA, USA). The analysis of blood metals was carried out as follows: the blood samples were digested in HNO_3_ and 30 % H_2_O_2_ and then analyzed using an Agilent 8800 ICP Triple Quad (ICP-QQQ) instrument (Agilent Technologies, Inc., Santa Clara, CA) in MS/MS mode. In addition, cord blood samples and the maternal venous blood sample were collected within 12 h after delivery. Details about laboratory procedures were previously published. (Kupsco et al., 2019; Renzetti et al., 2017) Two spot urine samples were collected during the second and third trimesters at the specified facility for the scheduled visit. These were preserved at −80 °C and subsequently sent to the Icahn School of Medicine at Mount Sinai. To determine the levels of metals in the urine, a 200 μL sample was diluted to 10 mL using a diluent solution comprising 0.5 % HNO_3_, 0.005 % Triton X-100, and a mixed internal standard. These dilutions were performed in polypropylene trace metal-free Falcon tubes. (Levin-Schwartz et al., 2021b) Quality control assays validated the precision and accuracy of these analytical procedures. (Braun et al., 2014) The study staff measured specific gravity to allow correction for urine dilution. We did data imputation of any missing specific gravity values, filling in the mean specific gravity for this sample for the same trimester. (Levin-Schwartz et al., 2021b; Wu et al., 2020) All urine measurements were adjusted by specific gravity correction method as follows:

$${\mathrm{Metal}}_{\mathrm{adjusted}}={\mathrm{Metal}}_{\mathrm{original}}\left(\frac{\mu SG\;-\;1}{SG\;-\;1}\right)$$

where ${\mathrm{Metal}}_{\mathrm{adjusted}}$ is the metal concentration adjusted for urine dilution, ${\mathrm{Metal}}_{\mathrm{original}}$, is the original metal concentration, *SG*, is specific gravity, and, *μSG*, is the mean specific gravity value for the subjects during the second (1.0163) and third trimester (1.0161).

The limit of detection (LOD) for blood metal levels ranged between 0.1 μg/dL and 0.7 μg/dL, the LOD for urinary metal levels ranged between 0.05 ng/mL and 0.2 ng/mL, for toenail samples was between 0.007 μg/g and 0.07 μg/g, and LOD for hair samples was 0.19 ng/g. Samples below the limit of detection (<0.01 %) were assigned a value of one-half the LOD per biomarker. Samples during the pre- and post-natal periods were primarily collected at scheduled clinic visits. Most samples were temporally clustered within a window, providing reasonable temporal consistency.

### 2.3. Childhood intellectual functioning assessment

The intellectual functioning of children was evaluated using the McCarthy Scales of Children’s Abilities (MSCA), a reliable and valid assessment tool broadly utilized in psychometrics that measures children’s cognitive and motor abilities between 2.5 and 8.5 years. (Backman et al., 1989; Kaufman and F. DiCuio, 1975; Levin, 2011; Teeter, 1984) The MSCA version employed in this study was translated from English to Spanish, it was standardized for Spanish-speaking children, and was only administered at an average age of 4.8 (±0.55) years, as part of a neurodevelopmental assessment. Trained psychologists at the National Institute of Perinatology conducted the assessment with direct interaction with children for approximately 60 min when they were 48 months old, encompassing various scales such as Verbal (assessing vocal expression and verbal comprehension), Perceptual Performance (evaluating visual-motor coordination and nonverbal reasoning), Quantitative (measuring understanding of quantitative concepts and numerical proficiency), and the General Cognitive Index (GCI) – a composite score that sums the verbal, perceptual performance, and quantitative scales. (Kippler et al., 2016) To adjust for age-related variation in performance, raw scores from the MSCA were transformed into age-standardized scores using a fractional polynomial approach, which allowed us to model smooth, age-specific reference ranges for cognitive outcomes. The General Cognitive Index was normalized to a mean of 100 ± 15. (Kupsco et al., 2020) Higher scores indicate higher cognitive function.

### 2.4. Covariate assessment

To identify potential confounders, we utilized directed acyclic graphs based on previous literature concerning covariates, and also calculated change-in-estimate (CIE) of 10 % or more according to our dataset. Those variables that fit this criterion were included in this study. In addition, those variables for this study were self-reported by the mothers during recruitment and follow-up with questionnaires. We finally considered maternal age (categorical), marital status (categorical), environmental tobacco smoke (categorical) by asking the exposure to second-hand smoking during pregnancy at home, socioeconomic status (categorical), trained psychologists assessed mother’s intellectual functioning (Log_2_) with the Wechsler Adult Intelligence Scale (WAIS) during the first month postpartum, mother education (categorical), breastfeeding (categorical), previous pregnancy (categorical), child education (categorical), child’s age at MSCA scores (continuous), and The Home Observation for Measurement of the Environment (HOME) score (continuous). The HOME score at 24 months postpartum was used to evaluate the quality and quantity of stimulation and support available to the child within the home environment. (Caldwell and Bradley, 2003)

### 2.5. Statistical approach

#### 2.5.1. Missing data

Some participants lacked complete data for our variables of interest (Tables S3 and S4). To include them in statistical models, we employed multiple imputations using chained equations through the MICE package in R, including the original PROGRESS sample of 1054 participants. During the imputation process, we considered the mother’s age at delivery, the child’s sex, the mother’s IQ levels, environmental tobacco smoke, socioeconomic status (SES), education, marital status, metal measurements, and health outcome. Our assumption was that the missing data occurred randomly, meaning that the absence of data was unrelated to any unobserved factors. As a result of this process, we generated 40 distinct imputed datasets. Following imputation, we retained participants who had both their intellectual functioning scores and at least one metal measurement (n = 604).

We modeled mother’s IQ levels and HOME scores in a way that best represented the shape of the association with intellectual functioning by assessing locally estimated scatterplot smoothing (LOESS) shape and by considering continuous, Log_2_, quadratic, five categories (percentiles 20, 40, 60, and 80) and four categories (quartiles), selecting the transformation with the lowest Akaike Information Criteria (AIC). (Vrieze, 2012) To prevent the influence of extreme values in our outcome measure, we winsorized (set extreme outliers equal to a specified percentile of the data) the GCI at the 1 and 99 percentiles.

#### 2.5.2. Multimedia biomarker to address high-dimensional measurement of exposure

Because each metal exposure was characterized via a number of measurements over time and across different media, it was not feasible to include them all in a model for our outcomes of interest. Instead, we chose to represent exposure over time to each metal via an integrated index that captured all prenatal and postnatal biomarker measures from different biologic tissues for each metal, which we termed a multimedia biomarker (MMB). Due to each chemical being distributed differently across the human body and across blood, urine, hair, and nails, each medium will provide distinct information about the true burden of each chemical in relation to neurodevelopment. MMBs address partial informative pathways by integrating information from different biomarkers into a single metric, with index weights that are selected to optimize the association with the health outcome, in this case – GCI. (Levin-Schwartz et al., 2020) We used the Weighted Quantile Sum Regression (WQS) method to create our MMBs, using 100 bootstrap datasets with weights for each input concentration (e.g. cadmium in 2^nd^ trimester blood) estimated as the average across the bootstrap estimates. Thus, MMB incorporates all the available biomarkers per metal across our time windows (e.g., BloodPb 2^nd^ trimester + BloodPb 3^rd^ trimester + UrinePb 2^nd^ trimester + UrinePb 3^rd^ trimester + ….+ Blood Pb 48 months) and calculated weighted sum with weights that sum 100 %. We considered different transformations of each biomarker (e.g. Log_2_ or quadratic transformations) and in general found that the original continuous variable exhibited adequate fit with the outcome variable, GCI, and so used the original variables to construct MMBs. Thus, we refer to MMB-metal (e.g, Pb, Cd, etc) as the relative contribution (weight) of metal to the overall WQS index in the context of its association with the GCI. The MMB score itself is a weighted index of the exposure biomarkers, created using the weights derived from their associations with the cognitive outcome. Hence, MMB-metal (e.g MMB-Cd) is a component or contributor to the overall WQS index, which reflects the weighted mixture exposure that best explains variation in the cognitive outcome. We also evaluated linearity by visually inspecting LOESS as described previously; the directionality of the association of the WQS index was constrained to be negative for non-essential metals and positive for those identified as essential in previous literature. The contributions of every biomarker in the index varied across every MMB (Fig. S3). While manganese is known to have a U-shape relationship to neurodevelopment (Claus Henn et al., 2010), normal urinary manganese concentrations typically range from 1 to 8 μg/L (Williams et al., 2012) However, given the narrow distribution and low levels observed in our study population (median: 1.4 μg/L; IQR: 1.3), manganese was classified solely as an essential metal. This decision reflects the likelihood that manganese exposure in this group is suboptimal rather than excessive. We also performed sensitivity analysis where we used the opposite assumption of direction when setting the MMB. While sample splitting is a cornerstone of many WQS implementations, (Carrico et al., 2015; Czarnota et al., 2015) we opted to follow an established methodology utilizing the MMB approach, which does not involve sample splitting. (Levin-Schwartz et al., 2020, 2021a, 2021b) This methodology has been utilized with an even larger sample size than ours. (Ashrap et al., 2021) Our analysis took this trade-off into careful consideration. However, splitting the sample would restrict each subset to a limited number of participants, leading to potential instability in weight estimation, diminished statistical power, increased variance in estimations, and could compromise the sensitivity and specificity of biomarker selection, critical factors in the realm of mixture models. (Day et al., 2022; Hargarten and Wheeler, 2020; Keil et al., 2020; Lyden et al., 2022) We elected not to divide our sample size, implementing 100 bootstrapped repetitions to stabilize the weights. However, we did a sensitivity analysis to evaluate the effect of not using split sampling under three conditions: a) using the full dataset, b) using a 50/50 split for training and validation, and c) inverting the training and validation sets. This approach aimed to examine the potential impact of overfitting, which may occur when the same data are used to both estimate component weights and evaluate the mixture-outcome association. By comparing models across these three conditions, we assessed the bias-variance trade-off inherent to WQS regression: full-sample models may yield overly optimistic estimates, while split-sample models reduce bias at the cost of increased variance and reduced statistical precision.

We employed generalized linear models for estimating associations between MMBs for each metal and child cognition, adjusting for maternal age, mother’s intellectual functioning, marital status, environmental tobacco smoke, socioeconomic status, education, child’s education, and HOME score, and in the final model, also including breastfeeding, previous pregnancy, and child’s age at the MSCA. In all models, we estimated the adjusted change in GCI scores according to an interquartile range (IQR) change in the MMBs using family gaussian and link function identity (Table S5).

Given that these metals share common sources, we also fit a model for estimating the associations with metals while adjusting for multi-pollutant confounding, (Braun et al., 2016) by simultaneously including all the MMBs:

$$E\left[GCI|\cdot \right]=\beta_{0}+\beta_{1}MMB_{1}+\beta_{2}MMB_{2}+\beta_{3}MMB_{3}+\ldots +\beta_{13}MMB_{13}+\beta_{x}\mathrm{confounders}$$

where GCI is the general cognitive index, $\beta_{0}$ is the intercept, MMB is the multimedia biomarker. To evaluate the impact of adjusting one metal for all others (co-pollutant confounding), we calculated the change in estimate after adjusting for other MMBs (relative to not adjusting for other MMBs).

Because previous studies showed the effect of some metals on cognition differed by child sex, (Anderson et al., 2012; Bauer et al., 2021; Lane et al., 2023) we assessed modification by child sex with the inclusion of an interaction term between each MMB and child sex in single-metal models, with an alpha level of 0.10. All the interaction models were adjusted by covariates. The statistical analyses were performed using RStudio (2023.06.0 + 421), and SAS 9.4 for interaction analysis.

## 3. Results

We found no substantial variations in the characteristics of the included subjects when compared to those who were excluded (Table S2). Among the included subjects, the median maternal age at delivery was 27.5 years (IQR: 7.96), with approximately 67 % of women being 30 years old or younger when giving birth. Half of the children were female (50 %), and nearly half (52 %) hailed from a low socioeconomic status background. The average children’s age at administering the MSCA was 4.81 ± 0.56 years old. The largest proportion of mothers (~60 %) had a high school education or higher, and 27 % of them practiced breastfeeding exclusively for 1 month. Participants were more commonly married (58 %) and did not have previous pregnancies (86 %). There were no considerable differences in any metal measure comparing those included and excluded (Table S2).

General cognitive index scores were positively associated with the mother’s IQ level, with up to an 8-point difference in mean GCI scores noted between the lower and higher quartiles of maternal IQ levels. Moreover, mothers with elevated socioeconomic status and education levels tended to have children with higher GCI scores. Those mothers with the highest IQ levels had less level of toxic metals. Thus, the MMB scores for lead, cadmium, arsenic, and barium ranged between 0.2 and 0.6 points of difference between moms with higher vs. lower IQ levels. We report a similar pattern by socioeconomic status and education, where those with higher socioeconomic levels had lower levels of lead and barium, while those moms with higher education had lower lead, cadmium, and cesium levels than their counterparts (Table 1).

**Fig. 1.**
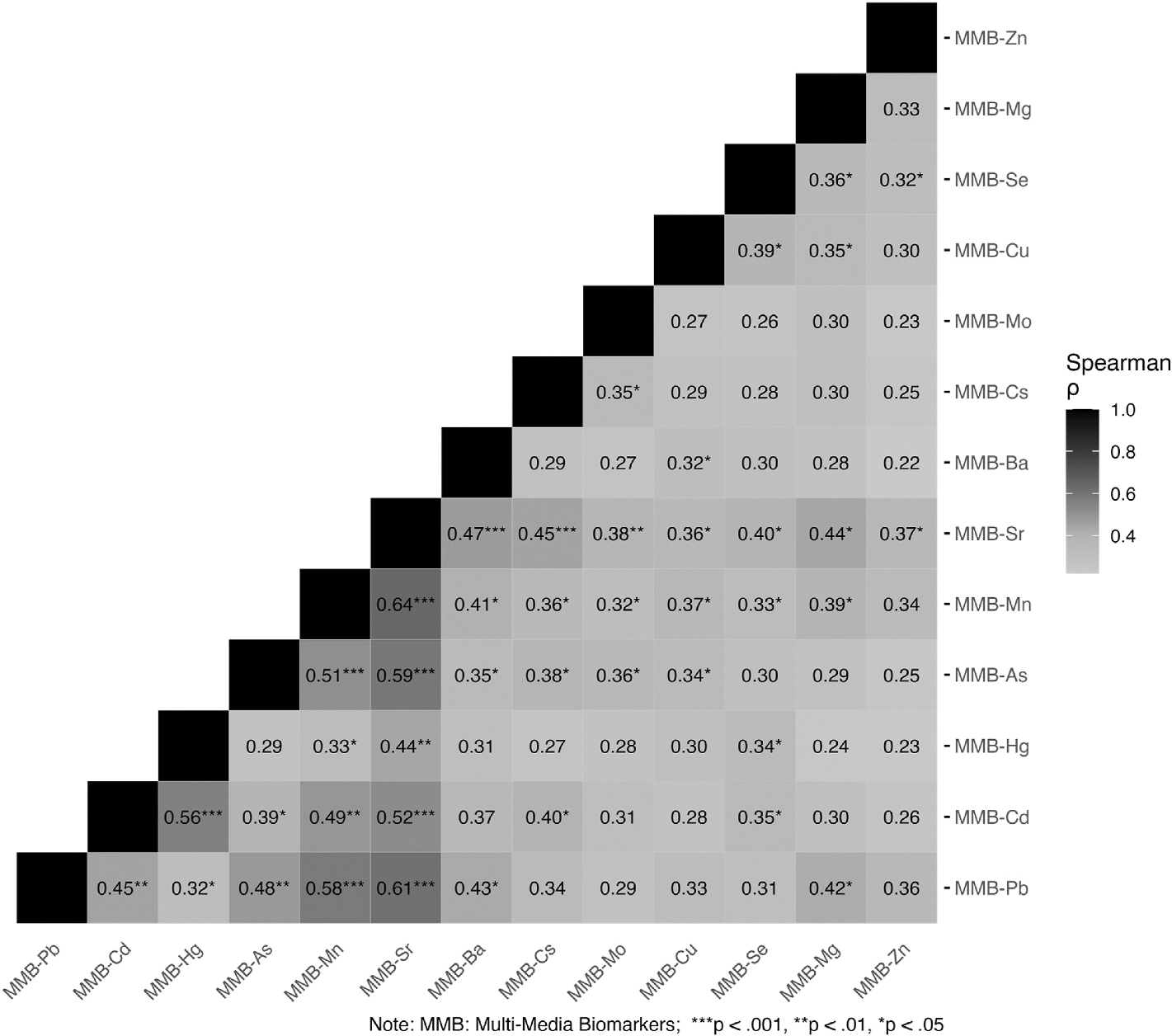
Spearman correlations between multimedia biomarkers.

We estimated Spearman correlations between each metal biomarker, ranging from the lowest value of −0.01 to 0.78 (Fig. S2). We also evaluated the correlation between MMB, finding that most correlations were weak. There were some stronger correlations between MMBs for essential elements, such as strontium and magnesium (r: 0.67) and strontium and molybdenum (r: 0.60) (Fig. 1).

After adjusting for confounders, we found that an interquartile increase in the lead MMB was associated with a 1.02-point decrease in the GCI, though the confidence interval included the null value (95 % CI: −2.51 to 0.47) (Table 2). Similarly, all non-essential metals exhibited adverse point estimates with the GCI, but all with confidence intervals spanning the null, with the largest found for arsenic (β: −1.40, 95 % CI: −2.81 to 0.02) and cadmium (β: −1.30, 95 % CI: −2.79 to 0.19). In contrast, essential metals were associated with increases in the GCI; with associations per IQR increase for manganese of 2.35 (95 % CI: 0.79 to 3.90), selenium of 2.31 (95 % CI: 0.92 to 3.68), and zinc of 1.84 (95 % CI: 0.28 to 3.38).

We found evidence that certain metals had co-pollutant confounded effects. When incorporated into multi-pollutant models, their point estimate changed because of the other metal under investigation (Table 3). Notable examples include the non-essential metal barium, where the measure of association was 0.83 GCI points stronger (more adverse) after adjusting for other metals (−1.40 (95 %CI: −2.98, 0.18) vs. −0.57 (95 %CI: −1.89, 0.76)). For the essential metal molybdenum, the association was 1.10 GCI points stronger (more beneficial) after adjusting for other metals (2.86 vs. 1.76). The association for zinc was weaker/attenuated after adjustment by 0.79 GCI points (from 1.13 to 0.34), although confidence bands for both zinc estimates included the null. Magnesium exhibited a large change, where the point estimate was negative after adjusting for other metals: −1.71 versus 0.06 in single-metal models, although confidence bands for both coefficients included the null (Table 4).

In our study, we didn’t find evidence that the effect of any of our MMBs on GCI differed by sex. Thus, among females, those with higher MMB scores didn’t have different effect sizes than males with higher MMB scores (Table 5).

In our sensitivity analysis, we evaluated impacts on our results of changing the direction of the constraint (positive or negative) for WQS when constructing each MMB. Here, the negative impact of lead was less robust after creating a MMB with the constraint that lead was positively associated with cognition. In contrast, the deleterious impact of mercury on cognition was evident even when we forced the creation of an exposure index (MMB) with the assumption that it supported cognition. Similarly, the fact that barium appeared to have an adverse relationship with cognition under two very different assumptions supports the neurotoxic effects of barium. In this population, manganese appeared to support cognition regardless of the assumption in our construction of the exposure index (MMB). In this sensitivity analysis magnesium exhibited an unexpected result, where it appeared to exert negative impacts on cognition after adjusting for other metal exposures. This negative influence persisted regardless of whether we built our exposure indices, assuming that magnesium was positively or negatively related to cognition (Tables S6 and S7). Finally, we replicated the regression models under three conditions, where in the full-sample models, we observed stronger associations with narrower confidence intervals. In contrast, both split-sample models yielded smaller effect estimates and wider confidence intervals than the full dataset. (Table S8)

## 4. Discussion

In this study, we leveraged all available data to construct a multi-media biomarker (index rather than relying on a single matrix. This approach also allowed us to include several understudied metals. We examined thirteen essential and non-essential metals in relation to child cognition at age 4 years, focusing on the independent effect of each of these metals after adjusting for all other non-essential metals. We have evidence that cadmium and arsenic had significant negative effects on child cognition, with lead and mercury also showing as adverse (but not reaching statistical significance). For essential metals, we found that molybdenum, and selenium had the strongest and significant positive effects on children’s general cognitive scores.

One ideal scenario in investigating metal exposures and intellectual functioning would be to identify those that have a real, independent detrimental or positive relationship. Toward this end, we adjusted each metal for all other included metals in multi-pollutant models to account for confounding due to shared sources, a current challenge in the field. (Braun et al., 2016; Kalkbrenner et al., 2018) We found evidence of such confounding patterns for some metals – both toxic and nutrient. For instance, in the group of non-essential metals, barium showed a negative effect on the general cognitive index, but after adjusting by other metals, it was more negative, possibly due to confounding by other metals. In our dataset, barium was correlated with strontium, magnesium, and molybdenum, which are also related to cognition. (Lai et al., 2021; Lu et al., 2023; Vaivada et al., 2017) Thus, these metals act as confounder factors for the relationship between barium and the general cognitive index. Another example of multi-pollutant confounding in this group of non-essential metals is cesium, which showed a negative effect on the general cognitive index in single pollutant models, and after adjustment by other metals, the effect was less negative. The strongest correlations for this metal were for cadmium and arsenic, both recognized as potent neurotoxicants. (Calderón et al., 2001; Egwunye et al., 2023; Gustin et al., 2018; Kippler et al., 2012)

Magnesium exhibited an unexpected negative effect on GCI in the multipollutant model, suggesting the possibility of co-pollutant confounding by metals such as strontium or molybdenum, which are correlated with magnesium, or simply that more extreme estimates can arise when adjusting for correlated exposures, a symptom of variance inflation. In addition, we encourage caution with these findings because highly correlated exposures may cause high variance, which can mean highly variable point estimates in addition to high variance estimates, which is a common finding in mixture analysis. Here, our results are in line with previous literature that associates both higher and lower magnesium levels with negative effects on children’s cognition. (Huang et al., 2019; Saghazadeh et al., 2017; Skogheim et al., 2021) It is necessary to be cautious and conduct more studies regarding this metal and its correlational pattern with other metals.

On the other hand, essential metals such as zinc, selenium, or copper are mainly beneficial in most cases, but sometimes may act detrimentally depending on their levels.(Florez-Garcia, 2024) Here, we need to indicate additional caution with manganese because it has been mainly evaluated as an essential metal with an inverted U-shaped effect on children’s cognitive outcomes. (Balachandran et al., 2020; Haynes et al., 2015) In our study, manganese was positively related to the GCI in both single and multi-pollutant models, which is still consistent with manganese playing a positive role in children’s cognition at early ages. In addition, an important portion of the scientific evidence for essential metals comes from experimental trials as nutritional supplements with positive results on cognitive outcomes, since they are historically identified as beneficial. (Gashu et al., 2015; Kessler et al., 2008; Khatry, 2012; Stoecker et al., 2009; Tamtaji et al., 2019) In this group of essential metals, we reported a negative effect of magnesium on children’s GCI yet included the null value, precluding the hypothesis of association. Most of the results from magnesium are positively related to cognitive outcomes; however, there is a similar finding where it was slightly related to negative effects in some of the cognitive domains such as quantitative, (Lai et al., 2021) which is part of the GCI. In adulthood, the results show that high magnesium intake is positively related to cognition in those people with appropriate serum vitamin D. (Tao et al., 2022) These discrepancies may be related to the media utilized. In our study, prenatal magnesium urine measures had the highest weight in our MMB; however, it is still challenging to have an accurate assessment of this metal, being usual to assess magnesium levels via serum blood. (Arnaud, 2008; S. Y. Kim et al., 2013; Rak et al., 2023) Another potential explanation for the negative effect of magnesium and GCI comes from its correlation pattern with strontium and barium.

Prenatal exposure to manganese (Mn), selenium (Se), and molybdenum (Mo) has been associated with enhanced neurodevelopment, potentially through mechanisms involving antioxidant defense, neurotransmitter regulation, and thyroid hormone synthesis. (Bowman et al., 2011; Keen et al., 2003; Rayman, 2012) These essential trace elements contribute to neuronal differentiation, synaptic plasticity, and protection against oxidative stress during critical periods of fetal brain development. In our study, the use of multi-matrix biomarkers (MMB) allowed for a more integrated estimation of cumulative exposures and minimized the potential for copollutant confounding, reinforcing the biological plausibility of their positive associations with cognitive function. Further, selenium’s role in neuroprotection and molybdenum’s involvement in key enzymatic reactions support their inclusion in mechanistic hypotheses on neurodevelopment. (Johnson et al., 1992) Continued research using mixture-based approaches will clarify the role of essential trace elements in shaping early cognitive outcomes.

Our results showed that the effect of our metals on children’s intellectual functioning does not differ by sex. This differs from some previous literature where some metals, such as urinary cadmium levels, were negatively associated with full-scale IQ and performance IQ in girls, but not in boys. (Zhou et al., 2020) Another study by Bauer et al. reported the sex impacts of manganese on cognition, with boys showing stronger positive associations. (Bauer et al., 2021) Rechtman et al. also reported that girls and boys are not equally vulnerable in terms of the effect of manganese and cognition. (Rechtman et al., 2023) Learning in boys is also more impacted by cadmium compared with their female counterparts. (Ciesielski et al., 2012) Thus, the current body of literature points to sex differences, but without a clear pattern about whether girls or boys are most impacted. Since we had a constrained sample size, this may have impacted our ability to detect interactions between metals and sex. Null interactions have also been pointed out in prenatal blood manganese levels and specific domains of children’s cognition. (Mora et al., 2018) We can set additional possible explanations for these discrepancies in two ways: a) the analyzed biomarkers, where we set an MMB as a more comprehensive estimation of the evaluated exposure, and b) the different time periods of neurodevelopment evaluated in the studies. Our MMB handled the exposure assessment in a different fashion, and we included pre- and post-natal periods. Furthermore, more research with a similar methodological approach will allow us to make more accurate comparisons regarding the heterogeneity analysis in very well-studied metals such as cadmium and arsenic. Nonetheless, in our stratified models, we observed that cadmium, arsenic, lead, and barium showed stronger negative associations with GCI among females, even though the sex-metal interaction terms were not statistically significant. This pattern suggests potential sex-related vulnerability that may still be of public health interest and deserves further exploration in larger samples.

In our study, cadmium and arsenic show a significant negative effect on GCI. Similar findings are reported by an important body of literature about these metals and their negative impacts on children’s cognitive outcomes. (Chatterjee and Kortenkamp, 2022; de Water et al., 2022; Heng et al., 2022; Skogheim et al., 2021; Yang et al., 2020) Since the correlation pattern with other metals may be different depending on the study area due to differing sources of metals, the co-pollutant confounding pattern may vary as well. For instance, in our study, the correlations of cadmium and other metals varied between −0.01 and 0.17, with cesium the strongest correlated; while in a study carried out in Arizona, New Mexico, and Utah, the correlations varied between −0.05 and 0.17 where manganese was the stronger one with cadmium. (Nozadi et al., 2022) Another study reported a correlation between cadmium and lead of 0.61.(Niehoff et al., 2021) In addition, that study shows a strong and positive correlation between arsenic and barium, which may be partially explained by the contamination of water sources via the utilization of hydraulic fracking from shale gas walls. Thus, the correlation of cadmium and arsenic with other metals will vary according to the study area, but it is still strongly related to detrimental effects on children’s cognition.

We report negative (but not statistically significant) effects of barium, an understudied metal on children’s intellectual functioning. Our findings are similar to other epidemiological evidence, where barium shows trends with having negative effects on children’s cognition, but in some cases without reaching statistical significance. (Nozadi et al., 2022; Thilakaratne et al., 2023; Tong et al., 2023) These findings could reflect that prior studies and our study lack the sample size to show significant impacts of barium. While it is possible that barium does not truly negatively impact child cognition, we note that the neurotoxic effects of barium are supported by some non-human evidence that barium competes with cellular potassium in nerve cells.(ATSDR, 2007) Taken together, we encourage further studies that analyze the effect of this metal on children’s intellectual functioning because we have evidence of its potential as a neurotoxicant at the epidemiological level. In the Mexican context, sources of barium include diet, drinking water, and pottery. (Peana et al., 2021) Barium is added to ceramics as barium carbonates to gain a matte tone in ceramics after adding it at low temperatures, (Vazquez, 2005) and thus pottery as a potential source of barium may require further attention.

The strongest correlation among our MMB were mainly limited to those between essential metals, where it is very well established that diet is the primary source. (Claus Henn, 2023) Zhou et al. (2020) in China and Nozadi et al. (2022) in the Navajo community in the U.S. showed different patterns of metal correlations compared with our findings, which highlight the fact that multimetal models are area-specific. Since correlations suggest shared sources, (Braun et al., 2016) the non-essential metals tested in our research may have different sources. For instance, of the total variability of lead exposure in Mexico, 87.3 % is explained by lead-glazed pottery usage, 4.2 % by other environmental sources, and 1.3 % by para-occupational exposures. (Trejo-Valdivia et al., 2023) As a result, conducting research in order to identify potential sources for these metals as individuals or mixtures of them is essential to better address the exposures. Particularly in the case of lead exposure, while the body of scientific literature about the negative impact of lead exposure on children’s cognitive outcomes is solid (Braun et al., 2012; Farías et al., 2022; Lanphear et al., 1998, 2005; Weitzman et al., 1993), our findings are mainly negatively associated with GCI but include the null value of association. Potential explanations for these findings may be that this population is immersed in a cohort that received counseling about toxic environmental pollutants from the recruitment, which may serve to buffer lead neurotoxicity. We want to emphasize that these findings are not definitive, being aware of the scientific evidence of lead as neurotoxic is extremely solid, and the important role of lead-glazed pottery in Mexican culture, where we encourage special caution about the translations of these findings. However, we also note that our findings underscore the challenges in identifying exposures that are independently associated with neurodevelopment among mixtures that contain toxic and salutary exposures.

We enhanced the robustness of our evidence by performing a sensitivity analysis allowing the construction of biomarker indices (MMB) assuming the opposite direction of impact on cognition from prior evidence. We found that for some metals, we have more definitive evidence that they are harmful (e.g., mercury, barium, strontium). For others, the evidence was mixed in terms of whether they are harmful or beneficial (e.g., cesium, copper, selenium), suggesting weaker evidence for a true causal influence of these metals on children’s cognition. Prior literature about environmental epidemiology and the role of some metals, such as strontium, molybdenum, and cesium, is very sparse and often performed in studies with modest sample sizes. (Levin-Schwartz et al., 2019; Liu et al., 2022; Nozadi et al., 2022) We stress, however, that this sensitivity analysis represents a very strong skepticism about the impacts of these exposures because they essentially make an assumption that opposes prior literature.

Upon deeply analyzing the split datasets, we observed a decrease in the strength of association and adjustment of the confidence intervals. This pattern aligns with the theoretical trade-off between bias and variance in predictive modeling: training and validating on the same dataset may introduce upward bias in effect size estimates due to overfitting, while sample splitting reduces such bias at the expense of statistical precision. (Steyerberg et al., 2001) This is particularly relevant in WQS regression, where variable weights are estimated empirically from the training sample and then applied to a second-stage regression model. (Carrico et al., 2015) Our sensitivity analysis thus provides an illustration of this bias-variance trade-off. While the full-sample estimate may overstate the association, the split-sample models, despite wider confidence intervals, offer a more conservative assessment of the relationship between MMB-lead and GCI. These results strengthen our methodological justification for using the full dataset in the main models while transparently reporting potential limitations. This approach is supported in the environmental epidemiology literature as a way to explore the robustness of findings in the context of highly correlated exposures and limited sample sizes. (Czarnota et al., 2015)

Our approach has several strengths. An important feature is our use of multipollutant models, which allow us to estimate the effect of every pollutant while controlling for many other factors. Our study includes an important period of neurodevelopment, pregnancy and early childhood, where the neurodevelopmental system is very sensitive to environmental pollutants. Our design was unlikely to suffer from differential exposure measurement error, because the measurement of metals in our study followed standardized procedures independent of, and prior to, the child’s intellectual performance measure. Additionally, we were able to include multiple, objective biomarkers of exposure that were analyzed using the latest generation of laboratory techniques. Our approach treated pre- and postnatal exposures as a unified early-life window to reflect the cumulative nature of developmental vulnerability. While we did not explicitly model toxicokinetics, integrating across time points and media aligns with mixture methods that aim to capture combined burden rather than isolate individual exposure sources. (Taylor et al., 2016)

Our multi-media biomarker methodology brought the strength of allowing us to leverage all the available information to estimate an index based on several media instead of one, also combining across time periods. Based on this approach, we integrated the information of media that better represents the overall body burden by every metal, increasing the statistical power (Levin-Schwartz et al., 2020) and even better reflecting chronic exposures. (Hu et al., 1998) Also, in contrast with some very studied metals such as lead and cadmium where the recommended media are blood and urine, in some cases, such as barium, we do not have a gold standard media for the measurement most relevant to neurodevelopment. In this situation, the multimedia biomarker casts a wide net and may optimizes the ability to detect associations. (Taylor et al., 2016) Simultaneously, we acknowledge that this MMB approach forces the direction of the associations of the biomarkers rather than evaluating them in an independent context with the general cognitive index. (Carrico et al., 2015; Keil et al., 2020) We find this limitation manageable because we based our choice of direction of effect on previous literature. For instance, it is well established that lead is detrimental to neurocognitive outcomes in children, which is why we constrained the direction of our multimedia biomarker for lead as non-positive. Combining exposure biomarkers will reflect a more accurate estimation of the exposure in the body burden instead of a single biomarker. (Bergdahl and Skerfving, 2008; Grandjean et al., 2004) Similarly, when we designed MMB, the overall effect is weighted by the effects of individual components, and thus, it may underestimate the effect if there are periods of heightened susceptibility. Although variations across the windows for collecting samples were possible, all available biomarkers across media and timepoints to reflect a composite were tiny enough to demonstrate no major variations across time. In addition, the integrated exposure estimate, rather than relying on a single matrix or moment of exposure, allows us to better estimate an aggregate effect instead of measurements at single time-points. (Levin-Schwartz et al., 2020) Another limitation of our study is the substantial proportion of missing cognitive scores from the MSCA, with 42.4 % of data missing in the original cohort. To address this, we used multiple imputation, as baseline characteristics between participants with and without cognitive assessments were largely comparable. However, we acknowledge that imputing the outcome variable, rather than only covariates, can introduce bias, particularly if the missingness is related to unobserved factors associated with the outcome. (Sterne et al., 2009) Although the observed differences between included and excluded participants were minimal in our dataset, we recognize this as a potential source of bias and a limitation of our approach. Although the WQS model was not cross-validated due to limited sample size, we acknowledge the potential for overfitting and advise interpreting the results with caution regarding their generalizability. Future studies with larger samples should incorporate validation to strengthen the robustness of findings. Finally, the summary effects are not directly comparable since they each comprise different biomarkers. Thus, we encourage caution when analyzing MMBs including different biomarkers in every single period of time.

In conclusion, our study is consistent with previous evidence about the neurotoxic effects of metals of interest in public health, such as lead, cadmium, mercury, and arsenic, on intellectual functioning. We also report that some understudied non-essential metals, such as barium, may be a risk factor for decreased children’s intellectual functioning. We encourage further attention to barium and its health effects on domestic ceramic users in Mexico. In our study, manganese, selenium, and molybdenum were essential metals that improved the general cognitive index in children aged 48 months. We analyzed our metals in multipollutant models and found that multipollutant-confounding problems are a common issue that we need to handle in mixture analysis. Even though we find a null association between some metals and children’s intellectual functioning, our results need to be considered as a non-definitive conclusion in the field, given our modest sample size that may have limited our ability to detect effects.

## Supplementary Material

supplementary material

## Figures and Tables

**Table 1:** Characteristics of children and families enrolled in PROGRESS, Mexico City, by cognitive score and metal levels.

	GCI		Multi-Media Biomarker index (MMB)																									
			Pb		Cd		Hg		As		Mn		Sr		Ba		Cs		Mo		Cu		Se		Mg		Zn	
														
	Mean	SD	Me	IQR	Me	IQR	Me	IQR	Me	IQR	Me	IQR	Me	IQR	Me	IQR	Me	IQR	Me	IQR	Me	IQR	Me	IQR	Me	IQR	Me	IQR

**Mothers age at delivery (years)**																												
≤25	98.8	12.5	4.6	2.9	4.2	2.8	4.1	4.8	4.5	2.3	4.8	2.7	4.6	2.9	4.6	3.0	3.9	3.5	4.6	3.1	4.5	2.4	5.6	2.3	5.0	3.9	4.6	2.4
25 to 30	99.0	13.9	4.5	2.9	4.5	3.0	4.7	4.6	4.5	2.1	4.2	2.1	4.4	2.5	4.3	2.7	4.4	3.1	4.4	3.5	4.4	2.4	5.4	2.0	4.4	3.2	4.3	2.3
30 to 35	101.0	15.3	4.7	2.3	4.7	2.6	5.0	4.8	4.5	2.2	4.4	2.1	4.6	2.6	4.5	2.7	4.4	2.8	4.9	3.5	4.6	2.7	5.8	2.3	4.6	4.2	5.0	2.2
35+	99.5	13.1	4.4	2.8	5.1	2.9	4.2	4.4	4.1	2.2	4.7	2.1	4.4	2.8	4.3	2.3	4.8	2.7	3.5	4.0	4.5	2.2	5.2	2.3	3.9	3.4	4.3	1.9
**Child’s Sex**																												
Male	97.7	13.6	4.8	2.9	4.6	2.7	4.0	4.0	4.5	2.3	4.2	2.1	4.6	2.6	4.4	2.9	4.3	3.1	4.7	3.7	4.6	2.6	5.6	2.2	4.5	3.6	4.4	2.4
Female	101.0	13.5	4.3	2.6	4.5	3.0	5.1	4.8	4.4	2.2	4.8	2.3	4.4	2.9	4.5	2.6	4.4	3.3	4.3	3.3	4.4	2.4	5.4	2.3	4.6	3.8	4.5	2.1
**Mom IQ levels** ^a^																												
≤76	94.5	13.4	4.7	2.4	4.7	3.1	3.9	4.0	4.5	2.2	4.1	2.3	4.2	2.5	4.5	2.6	4.3	3.2	4.6	3.5	4.2	2.6	5.2	2.4	4.4	3.6	4.5	2.2
76 to 86	100.0	12.2	4.7	2.7	4.6	2.6	4.8	4.6	4.6	2.0	4.6	2.3	4.7	2.6	4.6	2.7	4.4	3.4	4.8	3.3	4.7	2.0	5.8	2.3	4.8	3.1	4.7	2.2
86 to 94	101.0	13.9	4.7	3.0	4.4	3.2	4.8	5.1	4.5	2.3	4.6	2.4	4.4	2.9	4.3	2.8	4.5	2.7	4.3	3.5	4.4	2.9	5.7	2.0	4.5	3.5	4.5	2.4
94+	103.0	13.9	4.3	3.0	4.1	2.7	4.9	4.8	4.2	2.2	4.9	2.1	4.6	2.9	4.2	2.8	4.5	3.5	4.3	3.6	4.5	2.5	5.4	2.3	4.5	3.7	4.2	2.5
**Childbirth weight (g)**																												
<2500	99.0	12.8	4.5	3.4	4.6	3.8	5.0	4.5	4.9	2.1	5.0	2.7	4.2	2.6	4.6	3.0	5.1	2.6	3.7	2.9	4.6	2.6	5.4	2.1	3.9	3.3	4.6	2.1
2500+	99.5	13.7	4.6	2.8	4.5	2.7	4.3	4.8	4.4	2.2	4.4	2.2	4.5	2.7	4.4	2.7	4.3	3.2	4.6	3.6	4.5	2.5	5.6	2.3	4.6	3.7	4.5	2.3
**Household smoking in pregnancy**																												
1+	99.5	12.6	4.7	2.4	4.6	2.7	3.9	5.4	4.2	2.4	4.4	2.3	4.3	2.8	4.5	2.6	4.1	3.2	4.3	3.3	4.3	2.6	5.6	2.0	4.1	3.5	4.8	2.1
No smokers	99.4	14.0	4.5	3.0	4.5	2.9	4.9	4.7	4.5	2.1	4.6	2.3	4.5	2.7	4.4	2.8	4.5	3.2	4.6	3.5	4.6	2.4	5.5	2.4	4.8	3.7	4.4	2.3
**Socioeconomic Status**																												
Low	97.6	13.5	4.8	2.6	4.6	2.7	4.1	4.9	4.5	2.1	4.7	2.1	4.4	2.7	4.4	3.0	4.3	3.2	4.6	3.2	4.6	2.5	5.5	2.1	4.5	3.6	4.5	2.3
Medium	101.0	13.3	4.3	2.6	4.4	2.8	5.0	4.6	4.3	2.2	4.2	2.6	4.7	2.7	4.6	2.7	4.4	3.4	4.6	3.9	4.5	2.4	5.5	2.3	4.7	3.6	4.5	2.2
High	104.0	14.0	4.2	3.6	4.7	3.1	4.3	4.3	4.5	2.3	4.0	1.7	4.6	2.6	4.0	2.0	4.7	2.8	3.8	2.8	4.3	2.4	5.9	2.1	4.4	4.1	4.3	2.5
**Education**																												
<High school	96.2	13.0	4.7	3.0	4.8	3.0	4.2	4.8	4.5	2.1	4.6	2.3	4.4	2.6	4.4	2.7	4.3	3.3	4.6	3.3	4.4	2.4	5.4	2.2	4.4	3.4	4.4	2.0
High school	98.8	13.5	4.7	2.5	4.5	2.8	4.8	4.9	4.2	2.4	4.4	2.6	4.5	2.6	4.6	3.1	4.6	3.1	4.6	3.5	4.5	2.7	5.4	2.4	4.8	3.3	4.5	2.4
>High school	106.0	12.6	4.1	2.7	4.3	2.9	4.8	4.6	4.4	1.8	4.2	1.6	4.7	2.4	4.4	2.3	4.1	3.1	4.0	3.7	4.6	2.4	5.8	2.2	4.6	4.0	4.4	2.3
**Breastfeeding** ^b^																												
Never	98.9	18.2	4.3	2.5	5.3	2.3	4.5	3.9	4.8	1.8	3.9	2.0	5.2	1.9	5.1	2.5	4.4	2.1	5.1	2.7	4.6	3.2	5.2	2.5	5.0	4.2	4.5	1.9
Occasionally	98.3	16.1	4.4	2.9	5.2	3.1	4.9	3.9	4.5	1.4	4.4	2.3	4.7	2.5	5.2	3.1	4.1	3.5	4.1	3.7	4.0	2.5	6.0	2.4	5.2	3.0	4.8	2.2
Frequently	99.9	12.3	4.7	2.6	4.4	2.8	4.7	5.0	4.5	2.2	4.6	2.2	4.5	2.6	4.4	2.8	4.2	3.2	4.6	3.6	4.6	2.4	5.6	2.2	4.5	3.3	4.5	2.2
Always	98.9	14.3	4.5	2.8	4.3	2.8	4.2	3.8	4.1	2.2	4.2	2.5	4.2	2.7	4.2	2.5	4.6	3.4	4.1	3.5	4.4	2.4	5.4	2.3	4.5	4.0	4.5	2.4
**Marital status**																												
Married	100.0	13.5	4.5	2.8	4.4	3.0	4.7	4.9	4.5	2.2	4.5	2.1	4.4	2.9	4.3	2.7	4.4	3.1	4.4	3.6	4.6	2.4	5.5	2.3	4.5	3.5	4.4	2.2
Free Union	97.2	14.9	4.8	2.6	4.7	2.6	3.8	4.4	4.3	2.0	4.2	2.4	4.3	2.6	4.7	2.8	4.4	3.4	4.5	3.4	4.4	2.5	5.6	1.9	4.5	4.3	4.8	2.4
Single	100.0	12.0	4.5	2.8	4.7	2.5	5.1	4.0	4.5	2.1	4.7	2.5	4.8	2.1	4.6	2.8	4.2	3.3	4.7	3.4	4.5	2.3	5.3	2.6	5.0	3.1	4.4	2.4
Separated	94.5	0.7	6.3	0.4	5.6	2.4	4.6	2.4	6.2	0.4	6.3	0.1	5.3	0.8	5.2	0.2	5.2	0.1	5.8	0.2	3.2	0.8	5.6	0.7	3.5	0.9	3.7	0.3
**Previous pregnancy**																												
Yes	101.0	14.1	4.7	2.7	4.4	2.7	4.2	4.8	4.3	2.0	4.5	2.3	4.5	2.5	4.4	2.8	4.0	3.4	4.5	3.3	4.4	2.8	5.6	2.3	4.8	3.7	4.3	2.7
No	98.5	13.3	4.5	2.8	4.6	2.8	4.5	4.7	4.5	2.3	4.5	2.2	4.4	2.8	4.4	2.7	4.5	3.1	4.6	3.6	4.6	2.3	5.5	2.3	4.4	3.6	4.5	2.1
**HOME score**																												
≤32	96.1	12.8	4.7	2.8	4.6	2.7	4.0	4.8	4.6	2.1	4.5	2.4	4.6	2.6	4.7	2.8	4.6	3.2	4.7	3.7	4.7	2.5	5.4	2.2	4.7	3.3	4.6	2.3
32+	103.0	13.6	4.5	2.7	4.5	2.9	5.0	4.7	4.2	2.3	4.6	2.1	4.4	2.8	4.1	2.5	4.1	3.2	4.3	3.3	4.3	2.5	5.8	2.4	4.4	4.0	4.4	2.2
**Child in preschool at 4 years of age**																												
Yes	100.0	13.6	4.5	2.7	4.5	2.8	4.7	4.8	4.4	2.2	4.5	2.3	4.5	2.6	4.4	2.8	4.3	3.2	4.6	3.6	4.5	2.5	5.6	2.3	4.6	3.6	4.5	2.3
No	95.9	13.3	4.8	3.0	5.4	2.4	4.1	3.9	4.6	1.8	4.0	2.2	3.9	2.6	4.6	2.1	4.9	3.0	4.2	3.0	4.4	2.4	5.3	2.4	4.5	3.8	4.2	2.7

a Splitted based on better fit on AIC/BIC.

b Never: Never breastfeed; Occasionally: Started but didn’t sustain, Frequently: Non-exclusive at 1 month, Always: Exclusive at 1 month. IQR: Interquartile range; Me: Median; SES: Socioeconomic status; Pb: Lead; Cd: Cadmium; Hg: Mercury; As; Arsenic; Mn:Manganese; Sr: Strontium; Ba: Barium; Cs: Cesium; Mo:Molybdenum: Cu: Copper; Se: Selenium; Mg: Magnesium; Zn: Zinc. GCI: General Cognitive Index.

**Table 2:** Single pollutant model evaluating the associations with children’s General Cognitive Index at 48 months.

	General Cognitive Index (n=604)					
	Model 1^a^		Model 2^b^		Model 3^c^	
	β (95 %CI)		β (95 %CI)		β (95 %CI)	

**Multi-Media Biomarker**						
**Constrained to be negative**						
MMB Lead	−1.95	(−3.53, −0.36)	−1.06	(−2.54, 0.42)	−1.02	(−2.51, 0.47)
MMB Cadmium	−2.98	(−5.05, −0.89)	−1.20	(−2.67, 0.27)	−1.30	(−2.79, 0.19)
MMB Mercury	0.24	(−1.58, 2.05)	−0.93	(−2.61, 0.75)	−0.96	(−2.65, 0.74)
MMB Arsenic	−2.29	(−3.81, −0.76)	−0.76	(−2.77, 0.04)	−1.40	(−2.81, 0.02)
MMB Strontium	0.10	(−1.40, 1.60)	0.08	(−1.30, 1.45)	−0.01	(−1.40, 1.38)
MMB Barium	−1.04	(−2.47, 0.39)	−0.57	(−1.88, 0.74)	−0.57	(−1.89, 0.76)
MMB Cesium	−1.04	(−2.65, 0.58)	−0.73	(−2.20, 0.75)	−0.70	(−2.19, 0.79)
**Constrained to be positive**						
MMB Manganese	2.57	(1.01, 4.12)	2.25	(0.77, 3.72)	2.28	(0.77, 3.77)
MMB Copper	0.41	(−1.16, 1.98)	0.41	(−1.16, 1.98)	0.83	(−0.63, 2.28)
MMB Selenium	2.65	(1.18, 4.12)	1.96	(0.60, 3.30)	2.07	(0.69, 3.43)
MMB Molybdenum	1.07	(−0.60, 2.75)	1.65	(0.11, 3.18)	1.76	(0.21, 3.31)
MMB Magnesium	−0.14	(−1.84, 1.56)	0.17	(−1.39, 1.73)	0.06	(−1.52, 1.63)
MMB Zinc	0.90	(−0.55, 2.36)	0.95	(−0.38, 2.28)	1.13	(−0.25, 2.51)

MMB: Multi-media biomarker; GCI: General Cognitive Index; 95 %CI; 95 % Confidence interval; n: Number of Observations Used;

a Model 1: Crude model;

b Model 2: Adjusted by mom age (categorical), Child sex (categorical), mom intellectual functioning (Log_2_), marital status (categorical), environmental tobacco smoke (categorical), socioeconomic status (categorical), education (categorical), HOME score (continuous), and child education (categorical).

c Model 3: variables in model 2 plus breastfeeding (categorical), Child’s age at McCarthy test (continuous), and previous pregnancy (yes vs. no). β coefficients and 95 %CIs across 40 multiple imputed datasets. Beta coefficients and 95 % confidence intervals represent the association between the combination of media (e.g: urinary, blood, etc) and general cognitive index at 48 months; the beta reflects the change in GCI per IQR increase in metal (IQR: Interquartile range, as in Table 2). GCI scores were winsorized at 1 and 99 percentiles in all models.

**Table 3:** Multi-pollutant models evaluating the association between metals and children’s General Cognitive Index at 48 months.

	General Cognitive Index (n=604)					
	Model 1^a^		Model 2^b^		Model 3^c^	
	β (95 %CI)		β (95 %CI)		β (95 %CI)	

**Multi-Media Biomarker**						
**Constrained to be negative**						
MMB Lead	−1.62	(−3.22, −0.01)	−1.05	(−2.56, 0.47)	−0.92	(−2.45, 0.61)
MMB Cadmium	−2.04	(−3.62, −0.46)	−1.37	(−2.85, 0.11)	−1.52	(−3.03, −0.01)
MMB Mercury	0.26	(−1.53, 2.05)	−0.90	(−2.57, 0.78)	−0.88	(−2.57, 0.80)
MMB Arsenic	−2.28	(−3.87, −0.68)	−1.48	(−2.96, 0.00)	−1.53	(−3.02, −0.03)
MMB Strontium	1.39	(−0.86, 3.65)	0.49	(−1.59, 2.57)	0.34	(−1.75, 2.43)
MMB Barium	−2.06	(−3.76, −0.36)	−1.42	(−3.00, 0.15)	−1.40	(−2.98, 0.18)
MMB Cesium	−0.47	(−2.12, 1.18)	−0.34	(−1.87, 1.18)	−0.28	(−1.82, 1.27)
**Constrained to be positive**						
MMB Manganese	2.75	(1.20, 4.29)	2.43	(0.93, 3.92)	2.44	(0.92, 3.95)
MMB Copper	0.11	(−1.55, 1.77)	0.33	(−1.21, 1.87)	0.49	(−1.05, 2.04)
MMB Selenium	2.71	(0.96, 4.45)	1.76	(0.14, 3.38)	1.74	(0.10, 3.36)
MMB Molybdenum	1.99	(−0.11, 4.09)	2.56	(0.61, 4.50)	2.86	(0.89, 4.82)
MMB Magnesium	−1.76	(−4.31, 0.78)	−1.40	(−3.75, 0.94)	−1.71	(−4.08, 0.65)
MMB Zinc	0.12	(−1.56, 1.80)	0.18	(−1.39, 1.75)	0.34	(−1.27, 1.94)

MMB: Multi-media biomarker with model assumptions on the direction (positive or negative) with GCI; GCI: General Cognitive Index; 95 %CI: 95 % Confidence interval; n: Number of Observations Used

a Model 1: Crude model (adjusted:metal by metal)

b Model 2: Adjusted by mom age (categorical), Child sex (categorical), mom intellectual functioning (Log_2_), marital status (categorical), environmental tobacco smoke (categorical), socioeconomic status (categorical), education (categorical), HOME score (continuous), and child education (categorical) and other metals.

c Model 3: variables in model 2 plus breastfeeding (categorical), Child’s age at McCarthy test (continuous), and previous pregnancy (yes vs. no). β coefficients and 95 %CIs across 40 multiple imputed datasets. Beta coefficients and 95 % confidence intervals represent the association between the combination of media (e.g: urinary, blood, etc) and general cognitive index at 48 months; the beta reflects the change in GCI per IQR increase in metal (IQR: Interquartile range, as in Table S2). GCI scores were winsorized at 1 and 99 percentiles in all models.

**Table 4:** Associations between multimedia biomarkers of metals and GCI comparing single pollutant models and co-pollutant models.

	General Cognitive Index (n = 604)				Change in estimate^c^
	Single pollutant Model^a^		Multipollutant Model^b^		
	β (95 %CI)		β (95 %CI)		

**Multi-Media Biomarker**					
**Constrained to be negative**					
MMB Lead	−1.02	(−2.51, 0.47)	−0.92	(−2.45, 0.61)	0.10
MMB Cadmium	−1.30	(−2.79, 0.19)	−1.52	(−3.03, −0.01)	−0.23
MMB Mercury	−0.96	(−2.65, 0.74)	−0.88	(−2.57, 0.80)	0.07
MMB Arsenic	−1.40	(−2.81, 0.02)	−1.53	(−3.02, −0.03)	−0.13
MMB Strontium	−0.01	(−1.40, 1.38)	0.34	(−1.75, 2.43)	0.35
MMB Barium	−0.57	(−1.89, 0.76)	−1.40	(−2.98, 0.18)	−0.83
MMB Cesium	−0.70	(−2.19, 0.79)	−0.28	(−1.82, 1.27)	0.42
**Constrained to be positive**					
MMB Manganese	2.28	(0.77, 3.77)	2.44	(0.92, 3.95)	0.17
MMB Copper	0.83	(−0.63, 2.28)	0.49	(−1.05, 2.04)	−0.33
MMB Selenium	2.07	(0.69, 3.43)	1.74	(0.10, 3.36)	−0.33
MMB Molybdenum	1.76	(0.21, 3.31)	2.86	(0.89, 4.82)	1.10
MMB Magnesium	0.06	(−1.52, 1.63)	−1.71	(−4.08, 0.65)	−1.77
MMB Zinc	1.13	(−0.25, 2.51)	0.34	(−1.27, 1.94)	−0.79

MMB: Multi-media biomarker with model assumptions on the direction (positive or negative) with GCI; GCI: General Cognitive Index; 95 %CI: 95 % Confidence interval.

a Model adjusted by mom age (categorical), mom intellectual functioning (Log_2_), marital status (categorical), environmental tobacco smoke (categorical), socioeconomic status (categorical), education (categorical), HOME score (continuous), child education (categorical), breastfeeding (categorical), Child’s age at McCarthy test (continuous), and previous pregnancy (categorical).

b Model adjusted by the same variables in the prior model plus all the metals. β coefficients and 95 %CIs across 40 multiple imputed datasets. Beta coefficients and 95 % confidence intervals representing the association between the combination of media (e.g: urinary, blood, etc) and general cognitive index at 48 months; the beta reflects the change in GCI per IQR increase in metal (IQR: Interquartile range, as in Table S2). GCI scores were winsorized at 1 and 99 percentiles in all models.

c Change in estimate was calculated as [multipollutant model-single pollutant model].

**Table 5:** Sex as a modifier of associations between multimedia biomarkers of metals and general cognitive index from single-pollutant models.

	*p*-value^a^	General Cognitive Index (n = 604)			
		Male^a^		Female^a^	
		β (95 %CI)		β (95 %CI)	

**Multi-Media Biomarker**					
**Constrained to be negative**					
MMB Lead	0.394	−0.46	(−2.40, 1.47)	−1.71	(−3.88, 0.45)
MMB Cadmium	0.105	−0.02	(−2.15, 2.10)	−2.40	(−4.38, −0.41)
MMB Mercury	0.677	−0.64	(−3.07, 1.79)	−1.34	(−3.61, 0.92)
MMB Arsenic	0.643	−1.12	(−3.06, 0.82)	−1.77	(−3.74, 0.20)
MMB Strontium	0.821	−0.20	(−2.18, 1.79)	0.12	(−1.75, 1.98)
MMB Barium	0.142	0.37	(−1.44, 2.19)	−1.55	(−3.40, 0.29)
MMB Cesium	0.510	−1.15	(−3.18, 0.89)	−0.16	(−2.26, 1.93)
**Constrained to be positive**					
MMB Manganese	0.785	2.47	(0.37, 4.56)	2.08	(0.09, 4.06)
MMB Copper	0.267	1.63	(−0.38, 3.64)	0.02	(−2.00, 2.03)
MMB Selenium	0.174	2.99	(1.09, 4.87)	1.15	(−0.73, 3.03)
MMB Molybdenum	0.391	2.34	(0.28, 4.40)	1.02	(−1.21, 3.25)
MMB Magnesium	0.573	−0.41	(−2.58, 1.76)	0.47	(−1.71, 2.65)
MMB Zinc	0.254	1.89	(0.04, 3.73)	0.37	(−1.54, 2.29)

MMB: Multi-media biomarker with model assumptions on the direction (positive or negative) with GCI; GCI: General Cognitive Index; 95 %CI: 95 % Confidence interval.

a *p*-value for interaction term MMB*sex.

a Model adjusted by mom age (categorical), mom intellectual functioning (Log_2_), marital status (categorical), environmental tobacco smoke (categorical), socioeconomic status (categorical), education (categorical), HOME score (continuous), child education (categorical), breastfeeding (categorical), Child’s age at McCarthy test (continuous), and previous pregnancy (categorical). β coefficients and 95 %CIs across 40 multiple imputed datasets. Beta coefficients and 95 % confidence intervals representing the association between the combination of media (e.g: urinary, blood, etc) and general cognitive index at 48 months; the beta reflects the change in GCI per IQR increase in metal (IQR: Interquartile range, as in Table S2). GCI scores were winsorized at 1 and 99 percentiles in all models.

## Data Availability

The data that has been used is confidential.

## Appendix A. Supplementary data

Supplementary data to this article can be found online at https://doi.org/10.1016/j.envres.2025.122323.
