# Supplementary material for: Essential and non-essential metals in children’s intellectual functioning: A multi-media biomarker approach from a Mexico City birth cohort
Source: Environ Res. Author manuscript; Available in PMC 2026 Mar 10. (PMC12973447; doi:10.1016/j.envres.2025.122323)
Supplement: supplementary material [file NIHMS2138030-supplement-supplementary_material.pdf]

----------**Supplemental Material**-------------

Essential Metals in Children's Intellectual Functioning: A Multi-Media Biomarker Approach from A Mexico City Birth Cohort

Victor A. Florez-Garcia[1, 2, 3*], Robert O. Wright[4], Alexander P. Keil[5], Martha M. Téllez-Rojo[6], Sandra Martínez-Medina[7], Guadalupe Estrada[8], Amy E. Kalkbrenner[1]

[1.] Joseph J. Zilber College of Public Health, University of Wisconsin-Milwaukee, Milwaukee, WI, 53211, United States of America
[2.] Division of Epidemiology and Biostatistics, School of Public Health, University of Illinois Chicago, Chicago, IL, 60612, USA
[3.] Department of Public Health. Universidad del Norte. Barranquilla, Colombia.
[4.] Department of Environmental Medicine and Public Health, Icahn School of Medicine at Mount Sinai, New York, New York, United States of America
[5.] Epidemiology Branch, National Institute of Environmental Health Sciences, National Institutes of Health, Department of Health and Human Services, Research Triangle Park, North Carolina, United States of America
[6.] Centro de Investigación en Nutrición y Salud, Instituto Nacional de Salud Pública, Cuernavaca, Morelos, Mexico
[7.] Department of Developmental Neurobiology, National Institute of Perinatology. Mexico
[8.] Department of Immunobiochemistry, National Institute of Perinatology, Mexico.

***Corresponding Author:** School of Public Health. Division of Epidemiology and Biostatistics. University of Illinois Chicago. 1603 W. Taylor St. Suite 989 SPHPI, Chicago, IL 60612. E-mail: victor8@uic.edu

Table of Contents

Supplemental Material 1

Essential Metals in Children's Intellectual Functioning: A Multi-Media Biomarker Approach from A Mexico City Birth Cohort 1

Table S1. Hierarchical Selection of Metals. 1

Table S2. Characteristics of Children and Families Enrolled in PROGRESS, Mexico City, by Inclusion Status. 2

Table S3. Variables included in multiple imputation (n=1054). 6

Table S4. Comparing characteristics of original data and imputed data (n=604) 7

Table S5. Multimedia Biomarker Distribution. 10

Table S6. Single metal associations with children's General Cognitive Index at 48 months with an inverted indication of direction. 11

Table S7. Multi-pollutant models evaluating the association between metals and children's General Cognitive Index at 48 months with an inverted indication of direction. 12

Table S8. Sensitivity analysis evaluating the association between MMB-Lead and GCI, while comparing the analysis in the entire dataset vs. the split dataset. 13

Figure S1. Flowchart showing definition of the study population. 14

Figure S2. Spearman correlations between metals. 13

Figure S3. Barplot of weight contributions of metals on multi-media biomarker index by media and time at measurement 14

Table S1. Hierarchical Selection of Metals.

Metal	Prenatal								Postnatal					1st selection	2nd selection		3rd Selection	Main source for human exposure
	2T				3T				0		12	24	48					
	Blood	Urine	Nails	Hair	Blood	Urine	Nails	Hair	type	Blood	Blood	Blood	Blood	Potentially In Mexican pottery[†]	# articles in pubmed (11/24/2023)[a,b]	Dev Neurotox	Correlation .7+ with some of the other metals previously selected	
Arsenic (As)	X	X		X	X	X		X	M	X	X	X	X		71	✅		Water
Cadmium (Cd)	X	X		X	X	X		X	C,M	X	X	X	X		68	✅		Food and cigarettes
Cesium (CS)	X	X			X	X			M	X			X		8	Unknown	✅	Food and water
Copper (Cu)	X	X			X	X			M	X			X		47	✅		Food
Manganese (Mn)	X	X		X	X	X		X	M	X	X	X	X		85	✅		Food and beverages
Lead (Pb)	X	X		X	X	X			C,M	X	X	X	X	✅	1542	✅		Ceramic
Selenium (Se)	X	X			X	X			M	X			X		50	✅		Food
Zinc (Zn)	X	X			X	X			C,M	X			X	✅	137	✅		Food
Magnesium (Mg)		X				X							X	✅	57	✅		Food
Strontium (Sr)		X				X							X	✅	4	Unknown		Food
Molybdenum (Mo)		X				X							X		7	Unknown	✅	Food
Barium (Ba)		X				X							X	✅	3	Unknown		Food
Mercury (Hg)		X	X			X	X								268	✅		Water

C: Cord blood; M: Mother blood; 2T: Second Trimester; 3T: 3rd Trimester: 3rd selection means those metals strongly correlated with some of the metals in selection 1 or 2.

[†] Viniegra G, Escobar R. El riesgo del saturnismo por la cerámica folklórica mexicana. Sal Pub Mex. 1966 Jan;8(1):69–77) - Vazquez, E. Materiales Cerámicos. Propiedades, Aplicaciones y Elaboración. Universidad Nacional Autónoma de México (2005). Arrevilllaga V, Solano P, Suárez M. Vidriado para cerámica tradicional libre de plomo [Tesis]. [Iztapalapa]: Universidad Autónoma Metropolitana; 2006 - Torres L. Desarrollo de vidriados sin plomo para piezas cerámicas de alfarería tradicional de la región de Tzintzuntzan a base de vidrio reciclado [Tesis]. [Morelia,Michoacán]: Universidad Michoacana de San Nicolás de Hidalgo; 2009.

[a] Short search strategy in Pubmed, we change every metal in every search as well as prenatal by postnatal: ((arsenic OR arsenic exposure OR arsenic exposures) AND (prenatal OR pre-natal OR pregnancy OR prepartum)) AND (cognition OR cognitive OR intelligence OR IQ OR Intelligence quotient OR intellectual OR child neurodevelopment)

[b] Complete search strategy in Pubmed, we change every metal in every search as well as prenatal by postnatal: (("arsenic"[MeSH Terms] OR "arsenic"[All Fields] OR (("arsenic"[MeSH Terms] OR "arsenic"[All Fields]) AND ("exposure"[All Fields] OR "exposure s"[All Fields] OR "exposured"[All Fields] OR "exposures"[All Fields] OR "exposuring"[All Fields])) OR (("arsenic"[MeSH Terms] OR "arsenic"[All Fields]) AND ("exposure"[All Fields] OR "exposure s"[All Fields] OR "exposured"[All Fields] OR "exposures"[All Fields] OR "exposuring"[All Fields]))) AND ("prenatal"[All Fields] OR "prenatally"[All Fields] OR "prenatals"[All Fields] OR "pre-natal"[All Fields] OR ("pregnancy"[MeSH Terms] OR "pregnancy"[All Fields] OR "pregnancies"[All Fields] OR "pregnancy s"[All Fields]) OR "prepartum"[All Fields]) AND ("cognition"[MeSH Terms] OR "cognition"[All Fields] OR "cognitions"[All Fields] OR "cognitive"[All Fields] OR "cognitively"[All Fields] OR "cognitives"[All Fields] OR ("cognition"[MeSH Terms] OR "cognition"[All Fields] OR "cognitions"[All Fields] OR "cognitive"[All Fields] OR "cognitively"[All Fields] OR "cognitives"[All Fields]) OR ("intelligence"[MeSH Terms] OR "intelligence"[All Fields] OR "intelligences"[All Fields] OR "intelligent"[All Fields] OR "intelligently"[All Fields] OR "intelligibilities"[All Fields] OR "intelligibility"[All Fields] OR "intelligible"[All Fields]) OR "IQ"[All Fields] OR (("intelligence"[MeSH Terms] OR "intelligence"[All Fields] OR "intelligences"[All Fields] OR "intelligent"[All Fields] OR "intelligently"[All Fields] OR "intelligibilities"[All Fields] OR "intelligibility"[All Fields] OR "intelligible"[All Fields]) AND ("quotient"[All Fields] OR "quotients"[All Fields])) OR ("intellectual"[All Fields] OR "intellectualism"[All Fields] OR "intellectually"[All Fields] OR "intellectuals"[All Fields]) OR (("child"[MeSH Terms] OR "child"[All Fields] OR "children"[All Fields] OR "child s"[All Fields] OR "children s"[All Fields] OR "childrens"[All Fields] OR "childs"[All Fields]) AND "neurodevelopment"[All Fields]))) AND ((excludepreprints[Filter]) AND (clinicaltrial[Filter] OR meta-analysis[Filter] OR multicenterstudy[Filter] OR observationalstudy[Filter] OR pragmaticclinicaltrial[Filter] OR randomizedcontrolledtrial[Filter] OR researchsupportamericanrecoveryandreinvestmentact[Filter] OR researchsupportnihextramural[Filter] OR researchsupportnihintramural[Filter] OR researchsupportnonusgovt[Filter] OR researchsupportusgovtnonphs[Filter] OR researchsupportusgovtphs[Filter] OR researchsupportusgovernment[Filter]) AND (humans[Filter]) AND (english[Filter] OR spanish[Filter]))

Table S2. Characteristics of Children and Families Enrolled in PROGRESS, Mexico City, by Inclusion Status.

	Total		Status			
			Included		Excluded	
	**n=1054**	**100%**	**n=604**	**100%**	**n=450**	**100%**
**Mothers age at delivery (years)**						
[Median, IQR]	*[27.5, 8.04]*		*[27.6, 7.96]*		*[27.5, 8.06]*	
Missing	98		0		98	
**Child´s Sex**						
Male	498	0.53	303	0.50	195	0.57
Female	450	0.47	301	0.50	149	0.43
Missing	106		0		106	
**Mother's IQ levels**						
[Median, IQR]	*[86.0, 18.0]*		*[85.0, 12.4]*		*[84.9, 12.9]*	
≤76	207	0.27	156	0.26	51	0.27
76 to 86	213	0.27	157	0.27	56	0.29
87 to 94	172	0.22	133	0.23	39	0.20
94+	189	0.24	143	0.24	46	0.24
Missing	273		18		255	
**Child's age at McCarthy test (years)**						
[Median, IQR]	*[4.8, 0.75]*		*[4.8, 0.75]*		*[4.5, 0.74]*	
Missing	446		1		445	
**Childbirth weight (g)**						
[Median, IQR]	*[3050, 598]*		*[3062, 572]*		*[3038, 659]*	
≤2500	107	0.11	55	0.09	52	0.15

2500+	841	0.89	552	0.91	289	0.85
Missing	106				106	
**Household smoking in pregnancy**						
At leats 1 smoker	338	0.32	182	0.30	156	0.35
No smokers	709	0.68	418	0.70	291	0.65
Missing	7		4		3	
**Socioeconomic status**						
Low	540	0.51	313	0.52	227	0.50
Medium	395	0.37	230	0.38	165	0.37
High	119	0.11	61	0.10	58	0.13
**Education**						
<High school	425	0.40	243	0.40	182	0.40
High school	377	0.36	219	0.36	158	0.35
>High school	252	0.24	142	0.24	110	0.24
**Breastfeeding**						
Never breastfeed	52	0.07	34	0.06	18	0.09
Attempted/started but didn't sustain	47	0.06	37	0.06	10	0.05
Non-exclusive at 1 month	469	0.61	349	0.61	120	0.61
Exclusive at 1 month	203	0.26	154	0.27	49	0.25
Missing	283		30		253	
**Marital status**						
Married	593	0.56	348	0.58	245	0.54
Free Union	263	0.25	142	0.24	121	0.27
Single	193	0.18	112	0.19	81	0.18
Separated	3	0.00	2	0.00	1	0.00
Divorced	2	0.00	0	0.00	2	0.00

	n	Median	IQR	n	Median	IQR	n	Median	IQR
**Previous pregnancy**									
Yes	151	0.14		87	0.14		64	0.14	
No	903	0.86		517	0.86		386	0.86	
**HOME score**									
[Median, IQR]	*[32.0, 8.0]*			*[32.0, 8]*			*[31.0, 8.3]*		
Missing	557			179			378		
**Child in preschool at 4 years of age**									
Yes	555	0.92		552	0.92		3	0.75	
No	46	0.08		45	0.08		1	0.25	
Missing	453			7			446		
	**n**	**Median**	**IQR**	**n**	**Median**	**IQR**	**n**	**Median**	**IQR**
**Blood Metals (µg/L)**									
Lead (µg/dL)	1051	3.13	2.56	604	3.30	2.74	447	2.93	2.35
Cadmium	1051	0.28	0.22	604	0.29	0.25	447	0.25	0.17
Arsenic	1051	0.95	1.01	604	1.07	1.46	447	0.85	0.50
Manganese	1051	17.94	6.97	604	18.46	6.63	447	17.04	7.73
Strontium	556	15.60	5.83	550	15.60	5.88	6	15.30	3.90
Barium	556	0.88	0.32	550	0.88	0.31	6	0.81	0.44
Cesium	1051	3.00	1.20	604	3.13	1.17	447	2.78	1.09
Molybdenum	556	0.69	0.25	550	0.69	0.25	6	0.75	0.25
Copper (mg/L)	1051	1.51	0.29	604	1.48	0.26	447	1.57	0.30
Selenium	1051	235.75	37.74	604	231.59	35.89	447	241.14	43.61
Magnesium (mg/L)	556	46.20	9.80	550	46.25	9.80	6	45.35	5.05
Zinc (mg/L)	1051	6.24	1.55	604	6.52	1.59	447	5.84	1.48
**Urinary Metals* (µg/L)**									
Lead	948	3.56	4.24	604	3.69	4.28	344	3.45	4.06

Cadmium	948	0.22	0.20	604	0.22	0.21	344	0.22	0.18
Mercury	948	1.10	1.23	604	1.13	1.32	344	1.07	1.14
Arsenic	948	15.35	13.07	604	15.35	14.40	344	15.35	10.47
Manganese	948	1.44	1.18	604	1.48	1.32	344	1.38	1.01
Strontium	948	159.73	119.47	604	160.04	129.05	344	159.27	103.67
Barium	948	3.95	3.95	604	4.12	4.42	344	3.83	3.58
Cesium	948	8.37	4.63	604	8.14	5.13	344	8.76	3.71
Molybdenum	948	44.83	31.42	604	44.30	33.43	344	46.67	28.79
Copper	948	15.28	11.80	604	15.95	13.81	344	14.39	7.48
Selenium	948	47.87	24.41	604	47.63	31.54	344	48.00	18.93
Magnesium (mg/L)	948	68.76	47.25	604	71.08	54.31	344	66.69	36.40
Zinc (mg/L)	948	1.10	1.29	604	1.08	1.31	344	1.14	1.25
**Hair Metals (ng/g)**									
Lead	1045	487.48	812.10	603	498.88	821.62	442	475.71	783.62
Cadmium	1045	15.24	38.95	603	16.92	42.49	442	12.21	31.80
Arsenic	1045	30.96	34.95	603	32.03	35.48	442	30.09	33.67
Manganese	1045	418.42	669.77	603	455.92	687.18	442	383.15	630.63
**Nails Metals (µg/g)**									
Mercury	876	0.16	0.14	536	0.16	0.15	340	0.16	0.14

*Urinary measurements adjusted by specific gravity; IQ: Intellectual quotient; SES: Socioeconomic Status; HOME: Home Observation for Measurement of the Environment Score; IQR: Interquartile Range

Table S3. Variables included in multiple imputation (n=1054).

	Missing values	
	Count	Percent
**Sociodemographic and life-style**		
Mothers age at delivery	98	9.3%
Child Sex	106	10.1%
Mother IQ	273	25.9%
Childbirth weight	106	10.1%
Household smoking in pregnancy	7	0.7%
Socioeconomic Status	0	0.0%
Education	0	0.0%
Breastfeeding	283	26.9%
Marital status	0	0.0%
Previous pregnancy	0	0.0%
HOME Score	557	52.8%
Child attending school at 48 months	453	43.0%
**Outcome**		
General Cognitive Index	447	42.4%
Perceptual	447	42.4%
Quantitative	447	42.4%
Verbal	447	42.4%

IQ: Intellectual functioning; HOME: Home Observation for Measurement of the Environment Score. Biomarkers per media and period also were included on the imputation process.

Table S4. Comparing characteristics of original data and imputed data (n=604)

	Status			
	Original data		Imputed data	
	n=604	100%	n=604	100%
**Mothers age at delivery (years)**				
[Median, IQR]	*[27.6, 7.96]*		*[27.6, 7.96]*	
Missing	*0*		*0*	
**Child´s Sex**				
Male	303	0.50	303	0.50
Female	301	0.50	301	0.50
Missing	0		0	
**Mother's IQ levels**				
[Median, IQR]	*[86.00, 18.00]*		*[86.00, 18.00]*	
Q1: ≤76	156	0.26	162	0.27
Q2: 76 to 86	157	0.27	163	0.27
Q3: 86 to 94	133	0.23	133	0.22
Q4: 94+	143	0.24	146	0.24
Missing	18		0	
**Childbirth weight (g)**				
[Median, IQR]	*[3062, 572]*		*[3062, 572]*	
≤2500	54	0.09	54	0.09
2500+	550	0.91	550	0.91
Missing	0		0	0.00
**Household smoking in pregnancy**				
At leats 1 smoker	182	0.30	184	0.30
No smokers	418	0.70	420	0.70
Missing	4			
**Socioeconomic Status**				
Low	313	0.52	313	0.52
Medium	230	0.38	230	0.38
High	61	0.10	61	0.10
Missing	0		0	
**Education**				
<High school	243	0.40	243	0.40
High school	219	0.36	219	0.36
>High school	142	0.24	142	0.24
Missing	0		0	

	Status			
	Original data		Imputed data	
	n=604	100%	n=604	100%
**Breastfeeding**				
Never breastfeed	34	0.06	44	0.07
Attempted/started but didn't sustain	37	0.06	42	0.07
Non-exclusive at 1 month	349	0.61	357	0.59
Exclusive at 1 month	154	0.27	161	0.27
Missing	30			
**Marital status**				
Married	348	0.58	348	0.58
Free Union	142	0.24	142	0.24
Single	112	0.19	112	0.19
Separated	2	0.00	2	0.00
Missing	0		0	
**Previous pregnancy**				
Yes	234	0.39	234	0.39
No	370	0.61	370	0.61
Missing	0		0	
**HOME score**				
[Median, IQR]	*[32.0, 8]*		*[32.0, 8.0]*	
Missing	179		0	
**Child attending school at 48 months**				
Yes	552	0.91	559	0.93
No	45	0.07	45	0.07
Missing	7			
**Outcome**				
**General Cognitive Index**				
[Median, IQR]	*[101.00, 18.25]*		*[101.00, 18.25]*	
Missing	0		0	
**Perceptual**				
[Median, IQR]	*[52.00, 11]*		*[52.00, 11]*	
Missing	0		0	
**Quantitative**				
[Median, IQR]	*[46.00, 12]*		*[46.00, 12]*	

		Status			
		Original data		Imputed data	
		n=604	100%	n=604	100%
	Missing	0		0	
**Verbal**					
	[Median, IQR]	*[50.00, 12]*		*[50.00, 12]*	
	Missing	0		0	

IQ: Intellectual functioning; IQR: Interquartile range; HOME: Home Observation for Measurement of the Environment Score. Biomarkers per media and period also were included on the imputation process.

Table S5. Multimedia Biomarker Distribution.

		Median	1st Q	3rd Q	IQR
**MMB**					
	MMB-Lead	3.07	2.26	4.37	2.12
	MMB-Cadmium	4.55	3.05	5.82	2.77
	MMB-Mercury	4.27	2.11	6.87	4.76
	MMB-Arsenic	4.46	3.40	5.59	2.19
	MMB-Strontium	4.46	3.14	5.84	2.70
	MMB-Barium	4.44	3.11	5.85	2.74
	MMB-Cesium	4.39	2.91	6.10	3.19
	MMB-Manganese	4.48	3.42	5.73	2.31
	MMB-Copper	4.52	3.27	5.76	2.49
	MMB-Selenium	5.53	4.40	6.65	1.12
	MMB-Molybdenum	4.55	2.80	6.32	3.52
	MMB-Magnesium	4.55	2.71	6.33	3.63
	MMB-Zinc	4.47	3.39	5.64	2.25

MMB: Multi-media biomarker. Q: Quartile; . IQR: Interquartile Range

Table S6. Single metal associations with children's General Cognitive Index at 48 months with an inverted indication of direction.

	General Cognitive Index (n=604)					
	Model 1┼		Model 2┼┼		Model 3┼┼	
	β (95%CI)		β (95%CI)		β (95%CI)	
**MMB**						
**Constrained to be positive**						
Lead	0.79	(-0.71, 2.29)	0.73	(-0.68, 2.13)	0.74	(-0.68, 2.15)
Cadmium	1.20	(-0.65, 3.05)	0.56	(-0.72, 1.83)	0.61	(-0.68, 1.90)
Mercury	1.91	(0.28, 3.54)	-0.66	(-2.21, 0.89)	-0.78	(-2.36, 0.79)
Arsenic	0.55	(-0.92, 2.03)	1.17	(-0.18, 2.52)	1.06	(-0.31, 2.44)
Strontium	0.48	(-0.91, 1.86)	0.20	(-1.06, 1.47)	0.14	(-1.15, 1.42)
Barium	-0.36	(-2.11, 1.39)	-0.05	(-1.65, 1.55)	-0.14	(-1.76, 1.48)
Cesium	1.17	(-0.40, 2.74)	1.11	(-0.34, 2.57)	1.18	(-0.29, 2.65)
**Constrained to be negative**						
Manganese	-0.18	(-1.62, 1.27)	0.83	(-0.50, 2.15)	0.95	(-0.40, 2.29)
Copper	-1.09	(-2.79, 0.61)	-0.29	(-1.85, 1.27)	-0.23	(-1.81, 1.36)
Selenium	-0.36	(-2.16, 1.44)	0.34	(-1.29, 1.97)	0.46	(-1.19, 2.11)
Molybdenum	-0.29	(-2.01, 1.43)	0.15	(-1.43, 1.72)	0.13	(-1.45, 1.70)
Magnesium	-0.82	(-2.44, 0.81)	-0.33	(-1.81, 1.16)	-0.49	(-1.98, 1.01)
Zinc	0.22	(-1.33, 1.77)	0.58	(-0.84, 1.99)	0.79	(-0.70, 2.27)

MMB: Multi-media biomarker with model assumptions on the direction (positive or negative) with GCI; GCI: General Cognitive Index; 95%CI; 95% Confidence interval; n: Number of Observations Used; ┼Model 1: Crude model; ┼┼Model 2: Adjusted by mom age (categorical), Child sex (categorical), mom intellectual functioning (Log_2), marital status (categorical), environmental tobacco smoke (categorical), socioeconomic status (categorical), education (categorical), HOME score (continuous), and child education (categorical) ┼┼┼Model 3: variables in model 2 plus breastfeeding (categorical), Child's age at McCarthy test, and previous pregnancy (yes vs. no). β coefficients and 95%CIs across 40 multiple imputed datasets. Beta coefficients and 95% confidence intervals represent the association between the combination of media (e.g: urinary, blood, etc) and general cognitive index at 48 months; the beta reflects the change in GCI per IQR increase in metal (IQR: Interquartile range, as in Table 2). GCI scores were winsorized at 1 and 99 percentiles in all models. Note: This table represent the opposite constraint of table 4 results

Table S7. Multi-pollutant models evaluating the association between metals and children's General Cognitive Index at 48 months with an inverted indication of direction.

	General Cognitive Index (n=604)					
	Model 1†		Model 2††		Model 3†††	
	β (95%CI)		β (95%CI)		β (95%CI)	
**MMB**						
**Constrained to be positive**						
Lead	0.51	(-1.05, 2.07)	0.57	(-0.90, 2.03)	0.62	(-0.85, 2.09)
Cadmium	0.80	(-0.60, 2.19)	0.47	(-0.85, 1.78)	0.50	(-0.83, 1.83)
Mercury	1.73	(-0.03, 3.50)	-1.30	(-3.00, 0.40)	-1.43	(-3.15, 0.29)
Arsenic	0.49	(-1.11, 2.09)	0.87	(-0.60, 2.34)	0.64	(-0.86, 2.13)
Strontium	0.96	(-0.89, 2.81)	0.43	(-1.26, 2.13)	0.39	(-1.32, 2.10)
Barium	-0.95	(-3.06, 1.15)	-0.57	(-2.49, 1.36)	-0.68	(-2.62, 1.26)
Cesium	0.94	(-0.88, 2.76)	1.18	(-0.51, 2.87)	1.28	(-0.42, 2.98)
**Constrained to be negative**						
Manganese	0.20	(-1.78, 1.38)	0.68	(-0.77, 2.13)	0.85	(-0.62, 2.31)
Copper	-1.59	(-3.43, 0.25)	-0.66	(-2.35, 1.03)	-0.58	(-2.29, 1.12)
Selenium	-0.76	(-2.76, 1.23)	0.07	(-1.76, 1.91)	0.16	(-1.69, 2.00)
Molybdenum	-1.01	(-3.16, 1.14)	-0.18	(-2.15, 1.79)	-0.04	(-2.03, 1.95)
Magnesium	-0.92	(-2.74, 0.90)	-0.44	(-2.10, 1.23)	-0.64	(-2.32, 1.04)
Zinc	0.44	(-1.30, 2.19)	0.30	(-1.31, 1.90)	0.46	(-1.21, 2.14)

MMB: Multi-media biomarker with model assumptions on the direction (positive or negative) with GCI; GCI: General Cognitive Index; 95%CI: 95% Confidence interval; n: Number of Observations Used; †Model 1: Crude model (adjusted:metal by metal) ; ††Model 2: Adjusted by mom age (categorical), Child sex (categorical), mom intellectual functioning (Log_2), marital status (categorical), environmental tobacco smoke (categorical), socioeconomic status (categorical), education (categorical), HOME score (continuous), and child education (categorical) and other metals †††Model 3: variables in model 2 plus breastfeeding (categorical), Child's age at McCarthy test, and previous pregnancy (yes vs. no). β coefficients and 95%CIs across 40 multiple imputed datasets. Beta coefficients and 95% confidence intervals represent the association between the combination of media (e.g: urinary, blood, etc) and general cognitive index at 48 months; the beta reflects the change in GCI per IQR increase in metal (IQR: Interquartile range, as in Table S2). GCI scores were winsorized at 1 and 99 percentiles in all models. Note: This table represent the opposite constraint of table 5 results.

Table S8. Sensitivity analysis evaluating the association between MMB-Lead and GCI, while comparing the analysis in the entire dataset vs. the split dataset.

	General Cognitive Index -GCI-					
	Model 1┼			Model 3┼┼		
	β (95%CI)		CLD	β (95%CI)		CLD
**Multi-Media Biomarker**						
**Constrained to be negative**						
MMB Lead (Original/Full dataset)	-1.95	(-3.53, -0.36)	3.2	-1.02	(-2.51, 0.47)	3.0
MMB Lead (50:50)	-1.32	(-3.44, 0.79)	4.2	-0.56	(-2.61, 1.48)	4.1
MMB Lead (Inverted)	-1.66	(-3.86, 0.54)	4.4	-0.002	(-2.23, 2.23)	4.5

MMB: Multi-media biomarker; GCI: General Cognitive Index; 95%CI; 95% Confidence interval; CLD: Confidence Limit Difference; ┼Model 1: Crude model; ┼┼Model 3: variables in model 2 plus breastfeeding (categorical), Child's age at McCarthy test, and previous pregnancy (yes vs. no). β coefficients and 95%CIs across 40 multiple imputed datasets. Beta coefficients and 95% confidence intervals represent the association between the combination of media (e.g: urinary, blood, etc) and general cognitive index at 48 months; the beta reflects the change in GCI per IQR increase in metal (IQR: Interquartile range, as in Table 2). GCI scores were winsorized at 1 and 99 percentiles in all models. 50:50 means we split our dataset 50% for validation and 50% for training, and then ran the same analysis inverted/switched.

Figure S1. Flowchart showing definition of the study population.

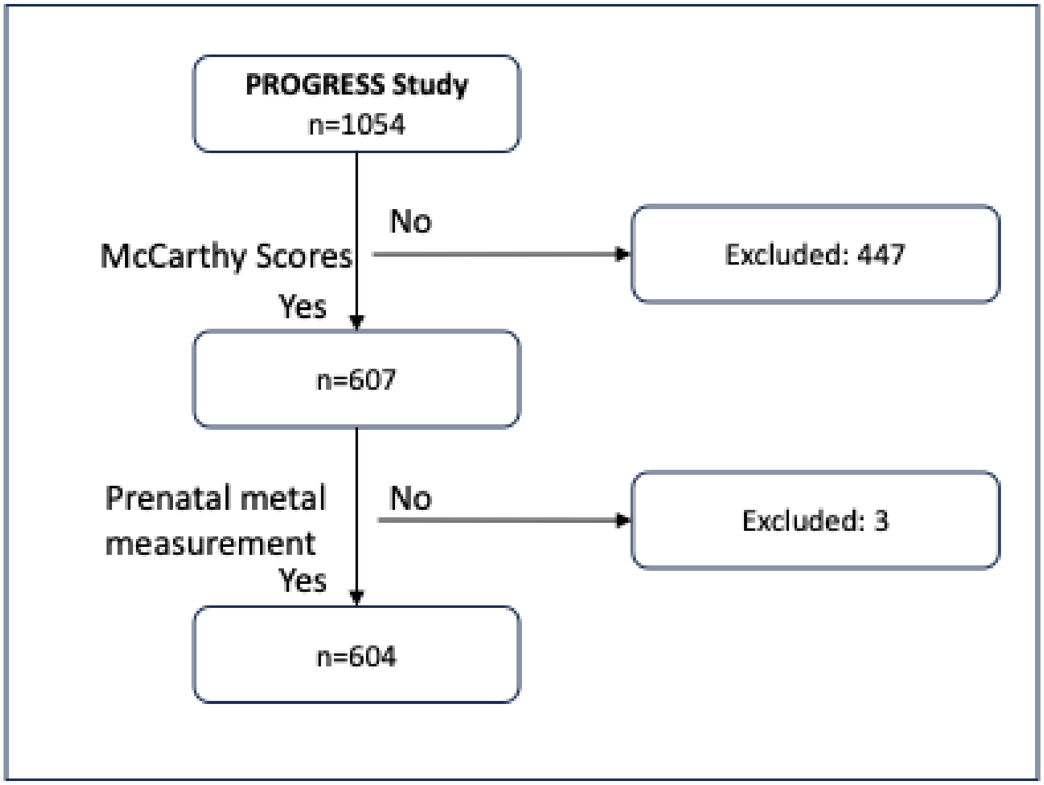

Figure S2. Spearman correlations between metals.

	URBa	URMg	URSr	URZn	URCd	URAl	URV	URCr	URMn	URCo	URNi	URCu	URAs	URSe	URMo	URSn	URSb	URCs	URHg	URTl	URPb	TNHg
**URBa**	1																					
**URMg**	0.50*	1																				
**URSr**	0.71*	0.75*	1																			
**URZn**	0.35*	0.40*	0.42*	1																		
**URCd**	0.35*	0.46*	0.46*	0.32*	1																	
**URAl**	0.48*	0.42*	0.43*	0.24*	0.43*	1																
**URV**	0.41*	0.54*	0.52*	0.40*	0.51*	0.53*	1															
**URCr**	0.15*	0.17*	0.15*	0.09*	0.22*	0.23*	0.25*	1														
**URMn**	0.55*	0.36*	0.39*	0.27*	0.44*	0.68*	0.55*	0.28*	1													
**URCo**	0.37*	0.56*	0.44*	0.31*	0.48*	0.44*	0.46*	0.19*	0.37*	1												
**URNi**	0.42*	0.44*	0.39*	0.33*	0.39*	0.54*	0.56*	0.32*	0.57*	0.54*	1											
**URCu**	0.49*	0.54*	0.52*	0.31*	0.61*	0.51*	0.61*	0.23*	0.57*	0.54*	0.50*	1										
**URAs**	0.36*	0.54*	0.49*	0.32*	0.45*	0.38*	0.59*	0.18*	0.38*	0.49*	0.42*	0.53*	1									
**URSe**	0.44*	0.69*	0.63*	0.48*	0.58*	0.47*	0.61*	0.25*	0.42*	0.54*	0.54*	0.62*	0.60*	1								
**URMo**	0.41*	0.72*	0.63*	0.39*	0.52*	0.42*	0.65*	0.23*	0.35*	0.53*	0.48*	0.56*	0.57*	0.75*	1							
**URSn**	0.40*	0.33*	0.30*	0.34*	0.27*	0.39*	0.42*	0.09*	0.45*	0.32*	0.33*	0.45*	0.36*	0.34*	0.30*	1						
**URSb**	0.46*	0.51*	0.49*	0.39*	0.43*	0.53*	0.69*	0.34*	0.53*	0.48*	0.63*	0.55*	0.48*	0.59*	0.57*	0.40*	1					
**URCs**	0.46*	0.71*	0.62*	0.46*	0.59*	0.47*	0.64*	0.24*	0.45*	0.56*	0.51*	0.62*	0.65*	0.78*	0.73*	0.38*	0.61*	1				
**URHg**	0.24*	0.41*	0.35*	0.27*	0.40*	0.29*	0.30*	0.08*	0.26*	0.34*	0.28*	0.38*	0.34*	0.47*	0.40*	0.24*	0.30*	0.48*	1			
**URTl**	0.45*	0.60*	0.56*	0.39*	0.55*	0.48*	0.56*	0.21*	0.44*	0.52*	0.47*	0.58*	0.58*	0.69*	0.63*	0.35*	0.53*	0.81*	0.43*	1		
**URPb**	0.41*	0.49*	0.51*	0.31*	0.43*	0.36*	0.45*	0.21*	0.35*	0.40*	0.37*	0.49*	0.44*	0.49*	0.48*	0.29*	0.43*	0.53*	0.28*	0.49*	1	
**TNHg**	-0.06	0.06	0.02	-0.0	-0.02	-3.74	-0.10	-0.0	-0.04	0.01	-0.05	0.00	0.00	0.01	-0.02	0.03	-0.05	0.03	0.21*	0.02	0.08*	1
**HCd**	-0.04	-0.03	-0.03	0.01	0.03	-0.03	-0.04	-0.0	-0.01	-0.03	0.01	-0.02	-0.05	0.00	-0.02	-0.03	0.01	-0.01	0.07	0.00	0.02	-0.02
**HMn**	-0.00	-0.04	-0.01	0.05	0.03	-0.00	-0.07	0.02	0.00*	-0.03	0.03	-0.00	-0.02	0.00	-0.02	-0.03	0.00	-0.05	0.04	-0.03	0.00	0.04
**HPb**	0.02	0.05	0.03	0.06	0.05	0.05	0.00	0.03	0.04	-0.01	0.00	0.07	0.00	0.02	0.06	0.02	0.04	0.05	0.05	0.04	0.15*	0.03
**HAs**	-0.02	-0.00	-0.02	-0.08	0.05	0.01	0.01	0.02	-0.00	0.04	0.04	0.02	0.03	0.04	0.03	-0.03	0.02	-0.00	-0.03	0.03	0.06	-0.04
**BPb**	-0.02	-0.01	-0.02	0.03	-0.00	-0.05	-0.04	0.03	-0.02	0.03	-0.02	-0.01	0.02	-0.08*	-0.03	0.01	-0.06	-0.02	-0.05	0.00	0.46*	0.07*
**BCd**	0.01	0.03	0.01	-0.11	0.14*	-0.00	-0.05	0.03	0.01	0.07	-0.03	-0.00	-0.02	-0.01	0.01	-0.04	-0.01	-0.00	0.00	0.00	0.00	0.05
**BAs**	-0.02	0.05	0.00	0.17*	0.04	0.02	0.03	-0.05	-2.43	0.06*	0.01	0.10	0.13*	0.08	0.02	0.08*	0.01	0.07	0.10	0.06	0.08	0.10*
**BCo**	0.00	0.05	0.01	-0.16*	0.05	0.05	0.01	-0.05	0.04	0.38*	0.03	0.02	0.08	-0.10*	-0.01	-0.02	0.00	-0.02	0.01	-0.02	0.01	0.06
**BCr**	-0.01	0.01	0.01	-0.10*	0.02	-0.00	-0.00	0.02	-0.00	0.03	-0.06	0.00	-0.01	-0.03	-0.04	0.02	0.03	-0.07	0.01	-0.07	0.01	0.01
**BCs**	-0.07	0.06	-0.06	0.12*	0.00	-0.01	-0.03	-0.05	-0.03	0.00	-0.08*	-0.06	0.09	-0.02	-0.00	0.11*	-0.03*	0.13	0.06	0.01	-0.03	0.16*
**BCu**	-0.02	0.01	-0.04	0.16*	-0.03	-0.05	-0.05	0.02	-0.07	-0.02	-0.03	-0.03	0.00	0.05	-0.01	0.04	-0.04	0.01	-0.00	-0.01	-0.03	0.04
**BMn**	-0.03	0.01	-0.04	0.16*	-0.01	-0.02	-0.03	-0.01	-0.02	0.09	-0.06	0.02	0.05	0.00	-0.06*	-0.02	-0.08*	0.00	-0.00	0.02	-0.00	0.05
**BSb**	-0.07	-0.06	-0.08	-0.16*	-0.08	-0.04	0.02	-0.05	-0.04	-0.06	-0.08	-0.01	0.05	-0.10*	-0.01	0.02	-0.02	-0.03	-0.02	-0.02	-0.05	0.00
**BSe**	-0.01	-0.03	0.02	0.15*	-0.03	-0.07*	-0.05	-0.06	-0.06	-0.07*	-0.08*	-0.10*	-0.02	0.01	-0.03	0.01	-0.07	-0.01	0.06	-0.04	0.02	0.08
**BZn**	-0.03	-0.02	-0.02	0.24*	-0.01	-0.05	-0.04	-0.04	-0.04	-0.00	0.00	-0.05*	0.00	0.06	0.00	0.09*	-0.02	0.01	0.07	0.03	0.00	-0.01
**BMg**	-0.06	-0.00	-0.04	-0.01*	-0.04	-0.02	-0.00	-0.05	-0.01	-0.05	-0.00	0.02	0.02	-0.02	0.00	0.04	-0.03	-0.04	0.02	-0.00	-0.01	0.01
**BAl**	0.08	0.00	0.07	-0.04*	0.03	0.04	0.04	0.01	0.09	0.09	0.05	0.08	0.07	0.02	0.04	-0.01	0.05	0.01	-0.03	0.01	0.06	-0.05
**BV**	-0.05	0.02	0.00	0.05*	-0.01	0.00	0.08	0.02	0.01	0.03	0.02	0.02	0.08	0.04	0.06	0.04	0.02	0.00	0.01	-0.01	0.02	-0.02
**BNi**	-0.05	0.01	-0.01	0.07*	-0.01	-0.02	-0.03	-0.02	-0.04	-0.01	-0.01	-0.03	0.01	0.03	0.04	-0.01	-0.05	0.01	0.04	0.04	0.03	0.02
**BMo**	-0.07	0.00	-0.06	-0.03*	-0.07	-0.03	-0.01	-0.02	-0.06	-0.04	-0.05	0.00	-0.02	-0.01	0.03	-0.02	-0.00	-0.03	0.01	-0.03	-0.07	0.06
**BSn**	0.10	0.03	0.07	0.00*	-0.01	0.04	0.03	-0.01	0.04	0.05	-0.01	0.00	0.12	-0.02	0.01	0.15	0.06	0.05	0.02	0.02	-0.02	-0.06
**BBa**	0.08	-0.01	0.06	0.07*	-0.02	-0.04	-0.04	0.02	-0.05	0.02	-0.01	-0.00	0.04	0.01	0.01	-0.04	-0.01	0.00	-0.04	0.00	0.07	0.05
**BTi**	-0.03	-0.01	-0.01	0.03*	-0.01	-0.00	0.02	-0.02	0.02	0.02	0.02	0.02	0.00	-0.00	-0.00	0.03	0.01	-0.03	-0.03	-0.04	-0.01	-0.01

	HCd	HMn	HPb	HAs	BPb	BCd	BAs	BCo	BCr	BCs	BCu	BMn	BSb	BSe	BZn	BMg	BAl	BV	BNi	BMo	BSn	BBa	BTi
**HCd**	1																						
**HMn**	0.48	1																					
**HPb**	0.55	0.51*	1																				
**HAs**	-0.04	-0.02	0.03	1																			
**BPb**	-0.04	0.04	0.10*	0.07*	1																		
**BCd**	-0.01	-0.07	0.00	0.04	0.01*	1																	
**BAs**	0.04	0.03	0.09	-0.00	0.07*	-0.28*	1																
**BCo**	-0.07*	-0.08*	-0.08*	-0.02	0.09	0.22*	-0.07*	1															
**BCr**	-0.01	-0.03	-0.07	0.04	0.03	0.04*	-0.04	0.19*	1														
**BCs**	-0.04	-0.13	0.00	-0.10*	0.07	0.03*	0.17*	0.05*	-0.01	1													
**BCu**	-0.00	0.06	0.07	-0.02	0.05*	-0.02	0.16*	0.02	-0.01	0.20*	1												
**BMn**	-0.00	0.01	0.00	-0.02	0.12*	-0.11*	0.42*	0.18*	-0.01	0.10*	0.25*	1											
**BSb**	-0.10*	-0.04*	-0.10*	-0.08	0.04	0.065	0.02*	0.20*	0.13*	0.16*	0.15*	0.06*	1										
**BSe**	0.03	0.01	0.03	-0.11*	0.06	-0.02*	0.19*	0.00	-0.01	0.22*	0.38*	0.25*	0.17*	1									
**BZn**	0.06	-0.01	0.04	-0.00	0.14*	-0.03*	0.22*	-0.06	-0.09	0.25*	0.41*	0.22*	0.09	0.52*	1								
**BMg**	0.00	0.01	0.00	-0.07	0.03	-0.03	0.02	-0.00	-0.04	0.07	0.10	0.03	0.25	0.27	0.17	1							
**BAl**	0.03	0.03	-0.01	0.03	0.05	0.013	0.01	0.05	0.14	-0.11	-0.20	-0.03	-0.00	-0.21	-0.14	-0.22	1						
**BV**	0.04	0.04	0.03	0.03	0.03	-0.01	0.10	0.05	0.00	0.05	0.14	0.09	0.16	0.16	0.09	0.41	0.04	1					
**BNi**	0.08	0.09	0.07	0.00	0.05	0.023	0.11	0.00	-0.02	0.05	0.19	0.16	0.13	0.20	0.25	0.30	-0.02	0.38	1				
**BMo**	-0.00	-0.00	-0.00	-0.01	-0.02	-0.00	0.05	0.13	-0.04	0.10	0.12	0.05	0.19	0.11	0.09	0.38	-0.13	0.33	0.16	1			
**BSn**	-0.03	-0.09	-0.09	-0.01	-0.06	0.044	-0.06	0.05	0.06	0.06	-0.0	-0.0	0.05	-0.03	0.03	0.10	0.08	0.10	-0.02	0.04	1		
**BBa**	0.05	0.09	0.01	-0.05	0.03	-0.05	0.11	0.06	-0.10	0.05	0.17	0.10	0.15	0.16	0.16	0.25	-0.00	0.33	0.33	0.18	0.04	1	
**BTi**	0.01	0.04	-0.03	-0.03	-0.04	-0.07	0.05	0.05	0.07	-0.01	0.01	0.02	0.00	-0.07	-0.08	0.08	0.20	0.16	-0.11	0.17	0.03	0.11	1

Note: Spearman correlations across the entire period of this study. *p<0.05

Figure S3. Barplot of weight contributions of metals on multi-media biomarker index by media and time at measurement.

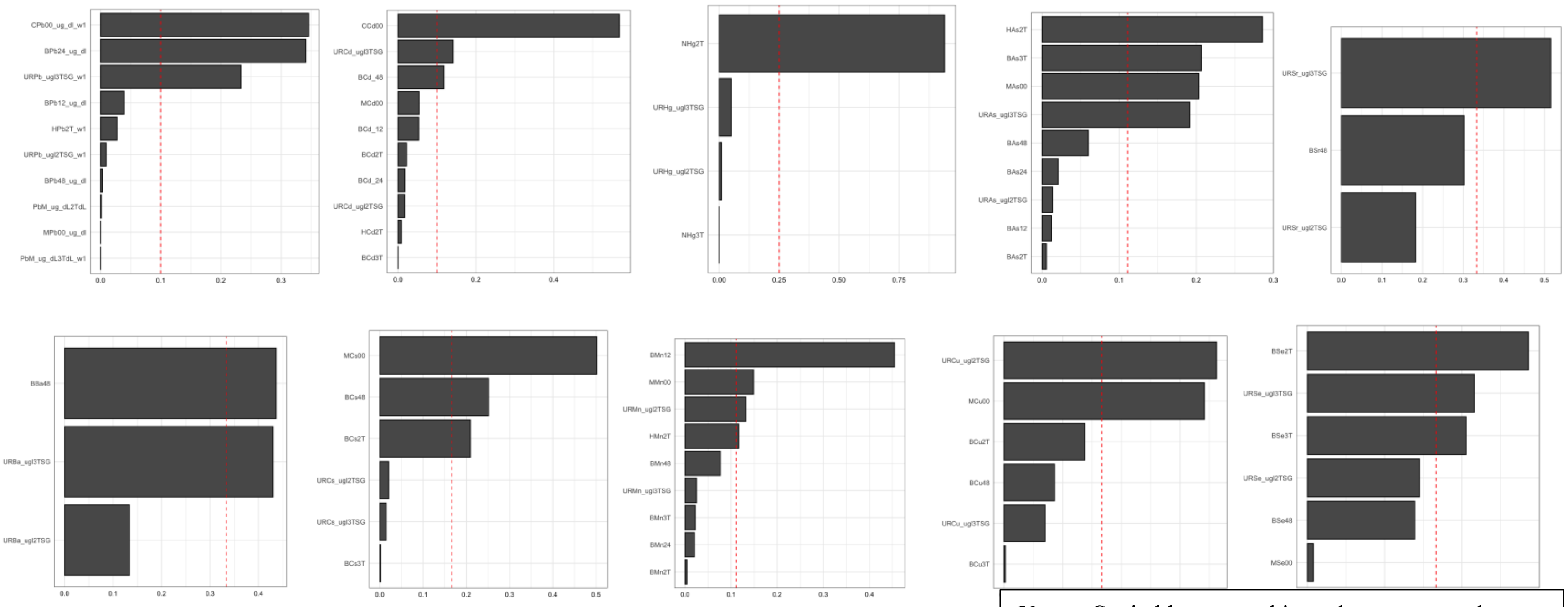

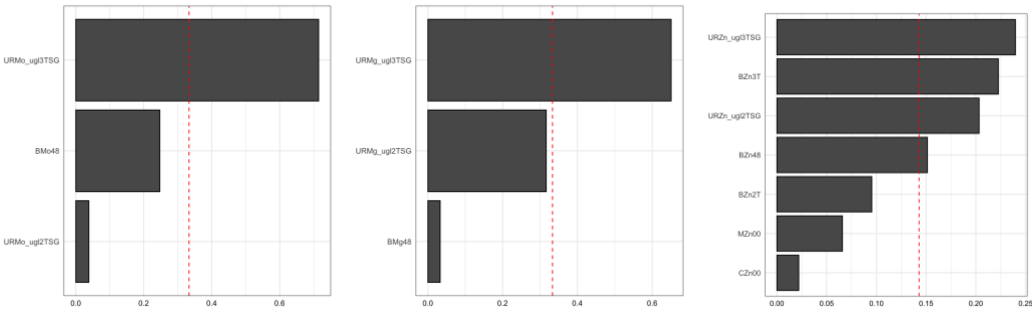

Notes: Capital letters per biomarker represent the original media, while numbers represent the pregnancy stage or the child's age. e.g., UPb3T means the urinary (U) lead in the third trimester (3T). Thus, C: cord blood; B: Blood; H: Hair; M; Blood from mom at delivery; 2T: second trimester; 3T third trimester; 00: Delivery; 12: twelve months after delivery; 24: twenty-four months after delivery; 48: forty-eight months after delivery.
